# m6A RNA methylation regulators were associated with the malignancy and prognosis of ovarian cancer

**DOI:** 10.1080/21655979.2021.1946305

**Published:** 2021-06-30

**Authors:** Cheng Zhang, JinHui Liu, Hongyu Guo, Dandan Hong, Jing Ji, Qin Zhang, Qun Guan, Qingling Ren

**Affiliations:** a Department of Gynecology, Jiangsu Province Hospital of Chinese Medicine, Affiliated Hospital of Nanjing University of Chinese Medicine, Nanjing, 210029 Jiangsu, China; b Department of Gynecology, The First Affiliated Hospital of Nanjing Medical University, Nanjing, 210029 Jiangsu, China

**Keywords:** Ovarian cancer, m6A, prognosis, immune cells infiltration, tcga

## Abstract

N6-methyladenosine (m6A) RNA methylation regulators play a regulatory role in tumor pathogenesis and development. However, the role of m6A regulator genes in ovarian cancer (OC) has not been fully elucidated. This study aims to investigate the mRNA expressions, clinicopathological features, and prognostic values of m6A regulators in OC. Here, we demonstrate that the 17 m6A RNA methylation regulators are differentially expressed in ovarian cancer and normal tissues. By using consensus clustering, all ovarian cancer patients can be divided into two subgroups (cluster 1 and 2) based on the expression of 17 m6A RNA methylation regulators. Using Gene Set Enrichment Analysis, we identified that cluster 1 was most connected to oxidative phosphorylation pathways. Regression models identified that prognosis is associated with HNRNPA2B1, KIAA1429, and WTAP. qRT-PCR result show that the expression trends of HNRNPA2B1 and KIAA1429 are consistent with the predicted results. Multivariate Cox regression analysis results show that the risk score was an independent predictive factor in OV. The overall survival of high-risk patients was significantly shorter than that of low-risk patients. ROC curve analysis showed that the prognostic signature precisely predicted the 5-year survival of OV patients. A nomogram was developed to predict each patient’s survival probability and well calibrated and showed a satisfactory discrimination. Dendritic fraction, macrophage fraction, and neutrophil fraction showed higher fraction in high-risk patients. In conclusion, m6A RNA methylation regulators are vital participants in ovarian cancer pathology, and three-gene mRNA levels are valuable factors for prognosis predictions.

## Graphical Abstract

**Figure:**
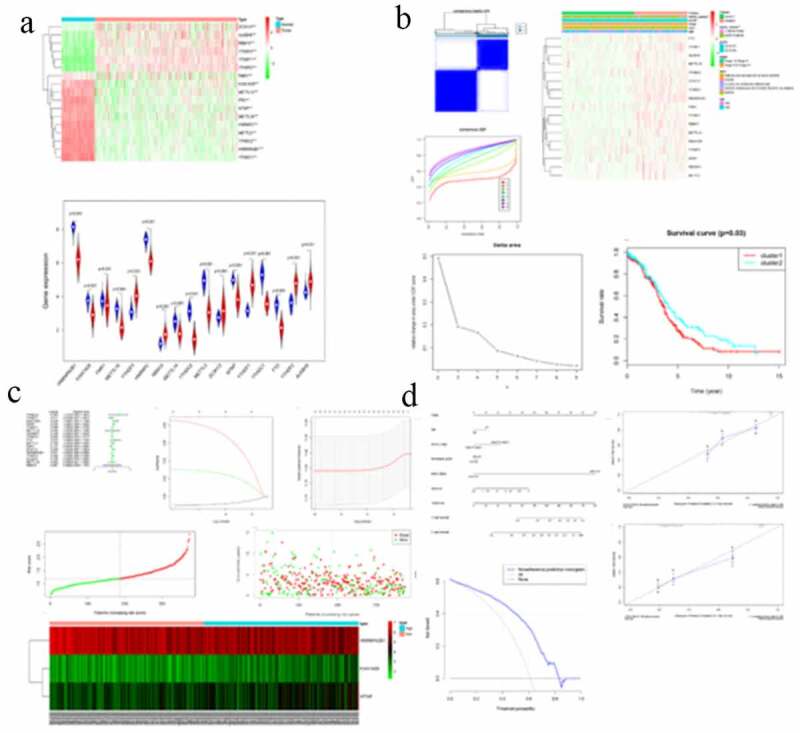

## Background

Epigenetic modification is a change in the expression of a nucleotide sequence. Previous studies have shed light on epigenetic pathways such as histone modification, chromosome remodeling, DNA methylation, and non-coding RNA regulation. RNA-level modification can be accomplished by N7-methyladenosine, N1-methyladenosine, pseudouridine, 5-methylcytosine, N6, 2ʹ-O-methylation, and 2ʹ-O-dimethyladenosine (m6A). Among them, m6A is a form of RNA methylation discovered in the 1970s.

RNA methylation is a dynamic and reversible process involving methyl-transferases ‘writers’, binding proteins (‘readers’), and demethylases (‘erasers’), just like DNA methylation. The prominent m6A methylation regulators include ‘writers’ like METTL3, METTL14, WTAP, KIAA1429, RBM15, and ZC3H13; ‘readers’ like YTHDC1, YTHDC2, YTHDF1, YTHDF2, and HNRNPC and ‘erasers’ like FTO and ALKBH5. More and more studies have found that regulators in m6A RNA methylation are associated with tumorigenesis. For example, Chen M et al. found that METTL3 promoted liver cancer progression [1]. Li J et al. found that FTO promoted the growth of lung cancer cells by regulating the m6A level of USP7 mRNA [2]. Hua W et al. found that METTL3 promoted ovarian carcinoma growth and invasion [3]. Mei Chen et al. found that m6A RNA methylation regulators can promote malignant progression and affect the prognosis of bladder cancer [4]. Shuai Ma et al. found that there is a meaningful interaction between m6A RNA methylation and non-coding RNA in cancer [5]. The mechanism of action between m6A RNA methylation and specific tumors has increasingly become the focus of in-depth research.

Molecular technologies for the treatment of ovarian cancer are ongoing. Ovarian cancer poses a great threat to women’s life. Abnormal modification of RNA may act in tumor development. The study by Zhao Ma et al. found that METTl3 independently of METT114 and WTAP regulates m6A in endometrioid epithelial ovarian cancer [6]. Jie Li et al. found that YTHDF2 is a protein inhibited by miR-145, which can regulate the proliferation, apoptosis, and migration of ovarian cancer cells [7]. Takeshi Fukumoto et al. found that N 6-Methylation of Adenosine of FZD10 mRNA Contributes to PARP Inhibitor Resistance [8]. These research results all show that m6A RNA methylation plays a pivotal role in the development and treatment of ovarian cancer.

The aim of our study is to find a direct and fixed algorithm for batch prediction of m6A RNA regulators that have an impact on ovarian cancer. Our goal is to provide a new molecular mechanism for the development of ovarian cancer.

## Materials and methods

### Data Sources

RNA-seq data and corresponding clinicopathological data were obtained from TCGA (http://cancergenome.nih.gov/http://cancergenome.nih.gov/) including 379 OC patients [9]. The expression dataset (N = 379 for ovarian cancer from TCGA and N = 88 for normal ovarian tissues from GTEx) [10] was downloaded from the UCSC Xena project (http://xena.ucsc.edu/http://xena.ucsc.edu/) [11]. Data were renormalized based on total reads for each sample to generate RPKM (Reads Per Kilobase of transcripts per Million mapped reads) [12] and then the expressions of between normal ovarian and OC tissues were compared. ‘limma’ package [13] was performed to solve the imbalance between the tumor and normal data and then analyzed different expressions among normal ovarian and OC.

Selection of m6A RNA methylation regulators

17 m6A RNA methylation regulators from published papers have been identified for subsequent studies.

### Bioinformatic analysis

We divided the samples into different groups using ‘ConsensusClusterPlus’ [14–16]. Enrich Database (https://amp.pharm.mssm.edu/Enrichr) was conducted for functional analysis. Interactions among m6A RNA methylation regulators were analyzed by using the Search Tool for the Retrieval of Interacting Genes Database (STRING) database (http://www.string-db.org/). Gene Set Enrichment Analysis (GSEA) [17] was based on different subgroups of OC for identifying the functions. Univariate Cox regression analysis [18] was used to determine the prognostic value of m6A RNA methylation regulators. From this, it has been proved that three genes were significantly associated with survival through this study (P < 0.05), which are selected for further functional analysis and development of a potential risk signature with the LASSO Cox regression algorithm [19]. The minimum criteria [20] was set to determine the 3 genes and their coefficients. The risk score [21] for the signature was calculated by using the formula: Risk score = ΣCoefi*xini = 1, where Coefi is the coefficient, and xi is the z-score-transformed relative expression value of each selected gene. This formula was used to calculate a risk score for each patient in TCGA datasets. To reveal potential Kyoto Encyclopedia of Genes and Genomes (KEGG) pathways [22] of the high- and low-risk groups and three prognosis-related genes, GSEA [23] was utilized to find enriched terms in C2 and in TCGA‐OV database. And *p* < 0.05 were considered to be statistically significant.

Complex cancer genomic profiles are accessible from the cBioPortal tool (http://www.cbioportal.org/http://www.cbioportal.org/) [24,25,].

The abundant six subtypes of tumor-infiltrating immune cells including CD4 T cells, CD8 T cells, B cells, neutrophils, macrophages, and dendritic cells can be visualized by TIMER online database [26].

### Construction and validation of the nomogram

A nomogram and calibration curve were constructed by using the ‘rms’ package [27] on R. Harrel’s concordance index (C-index) [28] was measured. The nomogram was then subjected to bootstrapping validation (1,000 bootstrap resamples) to calculate a relatively corrected C-index [29]. Finally, we used decision curve analysis (DCA) to determine the clinical usefulness [30].

### Statistical analysis

Patients were divided into two clusters by consensus expression of m6A RNA methylation regulators. Chi-square tests were used to compare the distribution of grade, race, stage age, and tumor status between the two risk groups.

Patients were divided into two groups based on the median levels of risk score [31]. This prognostic model and patient survival information were merged. Kaplan–Meier survival curves were used to compare the prognostic ability of the prediction models [32]. Area under the curve (AUC) [33] value for the Receiver operating characteristic (ROC) curves of each prognostic model was calculated by survival.ROC package in R. Besides, univariate and multivariate Cox regression analyses were conducted to compare the hazard ratio (HRs) [34] of prognostic models and important clinical features for OC. Differences among clinical parameters (age, grade, stage, and tumor status) were tested by using independent t-tests and *P* < 0.05 were considered to be statistically significant.

qRT-PCR to verify the expression of hub genes in clinical samples

16 ovarian cancer tissues and 8 normal ovarian tissues were obtained. The acquisition process and sample storage and processing are the same as before [35]. The extraction of RNA and the implementation of qRT-PCR were also operated strictly in accordance with the protocols. TABLE S1 showed the primer sequences.

## Results

The present study aims to identify m6A RNA modulators associated with prognosis in OC. We analyzed the association between each m6A RNA methylation regulator and the clinical features of OC. Survival analysis showed that ALKBH5, METTLE14, METTLE16, YTHDF1, YTHDF2, YTHDF3, and ZC3H13 were significantly associated. The expression profiles of METTLE3, YTHDC2, and YTHDF3 were all related to the clinicopathological features in OC. By using consensus clustering, all ovarian cancer patients can be divided into two subgroups (cluster 1 and 2) based on the expression of 17 m6A RNA methylation regulators. Using Gene Set Enrichment Analysis, we identified that cluster 1 was most connected to oxidative phosphorylation pathways. Regression models identified that prognosis is associated with HNRNPA2B1, KIAA1429, and WTAP. Combined with clinical characteristics, a nomogram for predicting prognosis was constructed and calibrated. The immune infiltration analysis was then performed to assess the impact of the three m6A RNA modulators on tumor behavior.

Expression of m6A RNA methylation regulators was correlated with OC clinicopathological features

**Figure 1.**
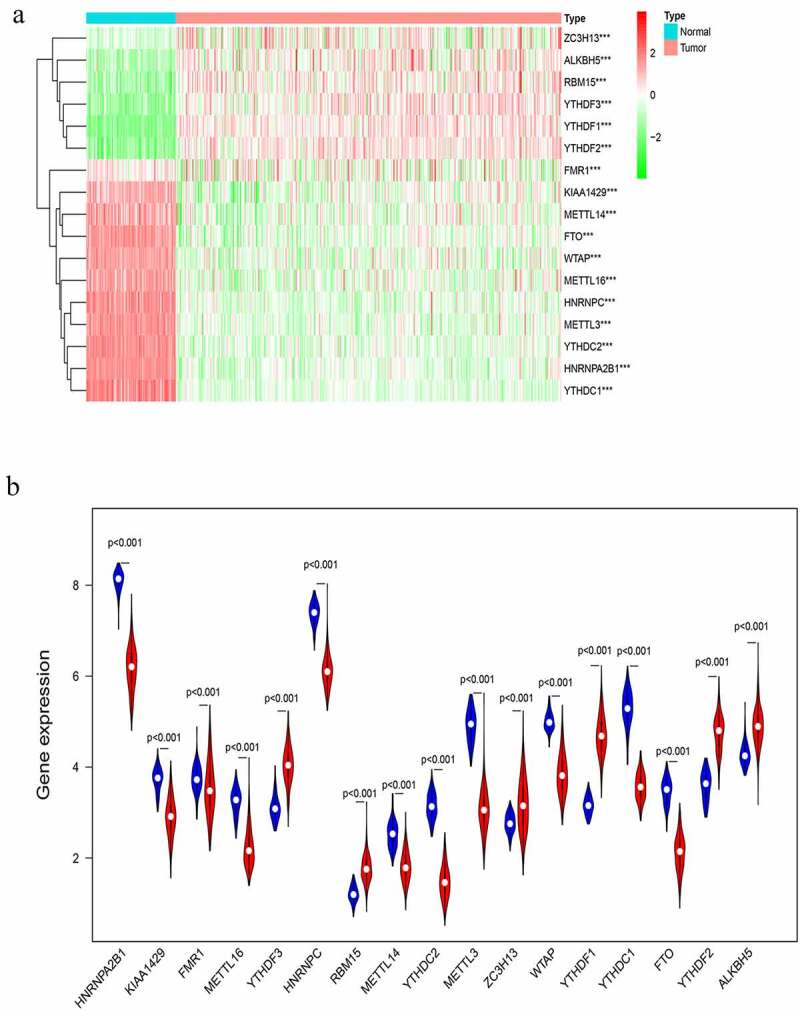
Expression of m6A RNA methylation regulator in OC samples and normal samples. (a) Heatmap showed that the 17 m6A RNA methylation regulators expressed differently between OC samples and normal samples. (b) Expression level of 17 m6A RNA methylation regulators in OC samples and normal samples

The correlation between each m6A RNA methylation regulator and OC clinical features was analyzed. These clinical features include age, grade, stage, and tumor-status. FMR1, METTL16, RBM15, and WTAP had significant correlations with age (**Supporting** Figure 1). FTO and YTHDC2 had significant correlation with OC grade (**Supporting** Figure 2). ALKBH5, METTL3, METTL14, METTL16, RBM15, and YTHDF1 had significant correlation with OC stage (**Supporting** Figure 3). HNRNPA2B1 and METTL16 had significant correlation with OC status (**Supporting** Figure 4). For ovarian cancer, BRCA1 gene mutation is a common pathogenic factor and marker for ovarian cancer [36]. The expression of the m6A RNA methylation regulator in ovarian cancer patients with and without BRCA1 mutation was also analyzed, and it has been found that the expression of both FMR1 and YTHDF1 showed differences between BRCA1-mutation and non-BRCA1-mutation groups (**Supporting** Figure 5). Seventeen m6A RNA methylation regulators in OC tissue samples and normal tissue samples were analyzed later, and it has been shown that all the regulators are differentially expressed (Figure 1). In addition, survival analysis found that the overall survival (OS) and progression-free survival (PFS) of ALKBH5, METTLE14, METTLE16, YTHDF1, YTHDF2, YTHDF3, and ZC3H13 were meaningful (**Supporting** Figure 6 **and Supporting** Figure 7). In order to assess the diagnostic value of 17 m6A RNA regulators, an ROC curve was generated by using expression data from ovarian cancer patients and healthy participants (**Supporting** Figure 8). The area under the ROC curve (AUC) indicates a modest diagnostic value.

**Figure 2.**
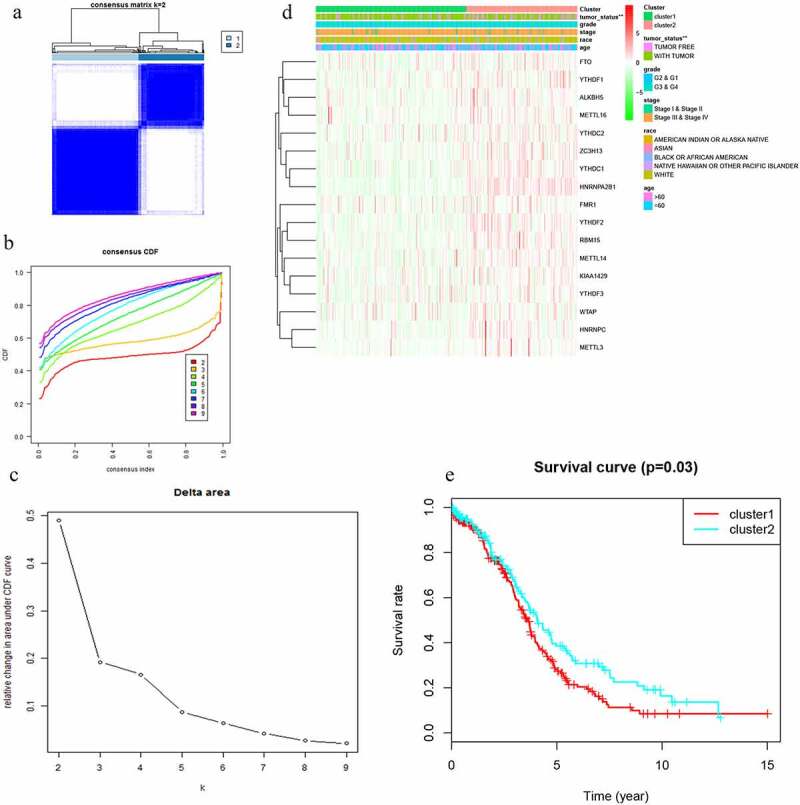
Differential clinicopathological features and overall survival of OC in the cluster ½ subgroups. (a) Consensus clustering matrix of 379 TGGA samples for k = 2. (b) Consensus clustering cumulative distribution function (CDF) for k = 2 to 10. (c) Relative change in area under CDF curve for k = 2 to 10. (d) Heatmap and clinicopathologic features of the two clusters defined by the m6A RNA methylation regulators consensus expression. (e) Kaplan–Meier overall survival (OS) curves for 315 out of 379 OC samples in the TCGA dataset

**Figure 3.**
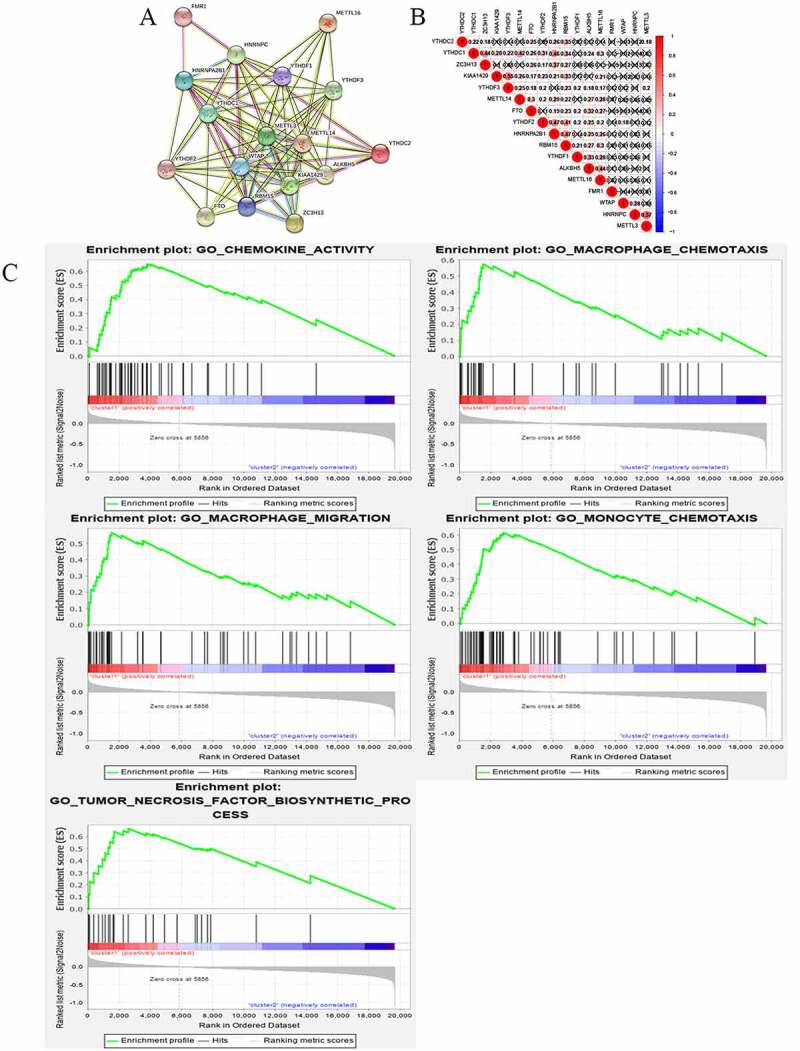
Interaction among m6A RNA methylation regulators and functional annotation of OC in cluster ½ subgroups. (a) The m6A modification-related interactions among the 17 m6A RNA methylation regulators. (b) Spearman correlation analysis of the 17 m6A modification regulators. (c) GO analysis by GSEA of cluster 1 and cluster 2

**Figure 4.**
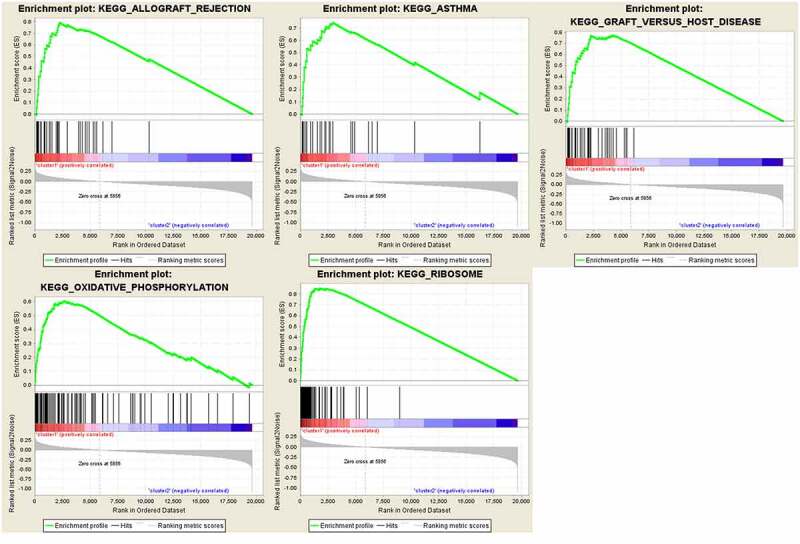
Gene set enrichment show genes with higher expression in cluster 1 were enriched for KEGG of malignant tumors

**Figure 5.**
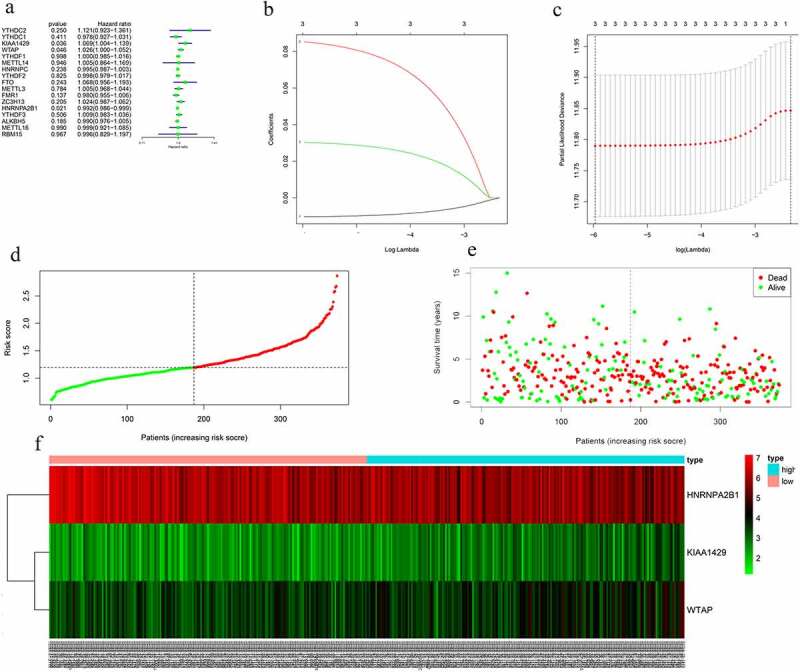
Risk signature with 17 m6A RNA methylation regulators. (a) The process of building the signature containing 17 m6A RNA methylation regulators. The hazard ratios (HR), 95% confidence intervals (CI) calculated by univariate Cox regression. (b) LASSO regression analysis was used to calculate the coefficient of interferon gamma response genes. (c) Three genes were selected as active covariates to determine the prognostic value after 10-fold cross-validation for the LASSO model. (d-e) The risk scores for all patients in TCGA cohort are plotted in ascending order and marked as low risk (blue) or high risk (red), as divided by the threshold (vertical black line). (f) The distribution of risk score, survival status, and the expression of 3 genes of each patient in TCGA cohort by z-score, with red indicating higher expression and light blue indicating lower expression

**Figure 6.**
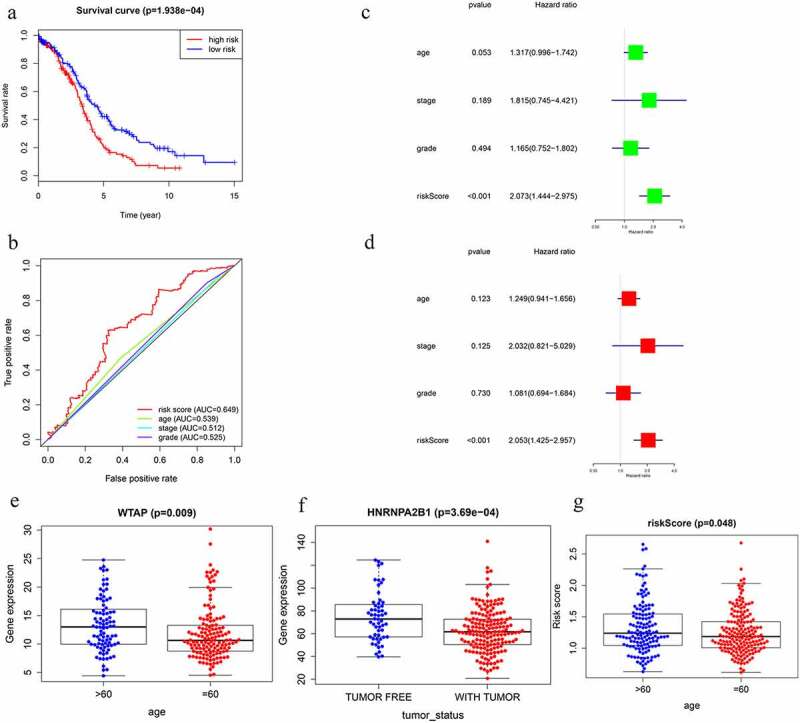
Screening of Prognosis-related m6A RNA methylation regulators. (a) Kaplan–Meier overall survival (OS) curves for patients in the TCGA datasets assigned to the low- and high-risk groups. (b) ROC curve for 5-year survival prediction and clinical characteristics, including age, stage, grade, and risk score. (c) Univariate Cox regression analysis of the associated between clinicopathological factors (including risk score) and overall survival of patients. (d) Multivariate Cox regression analysis of the associated between clinicopathological factors (including risk score) and overall survival of patients. (e) WTAP expression levels in different age groups. (f) The expression levels of HNRNPA2B1 in the TUMOR FREE group and the TUMOR group. (g) RISK SCORE in different age groups

**Figure 7.**
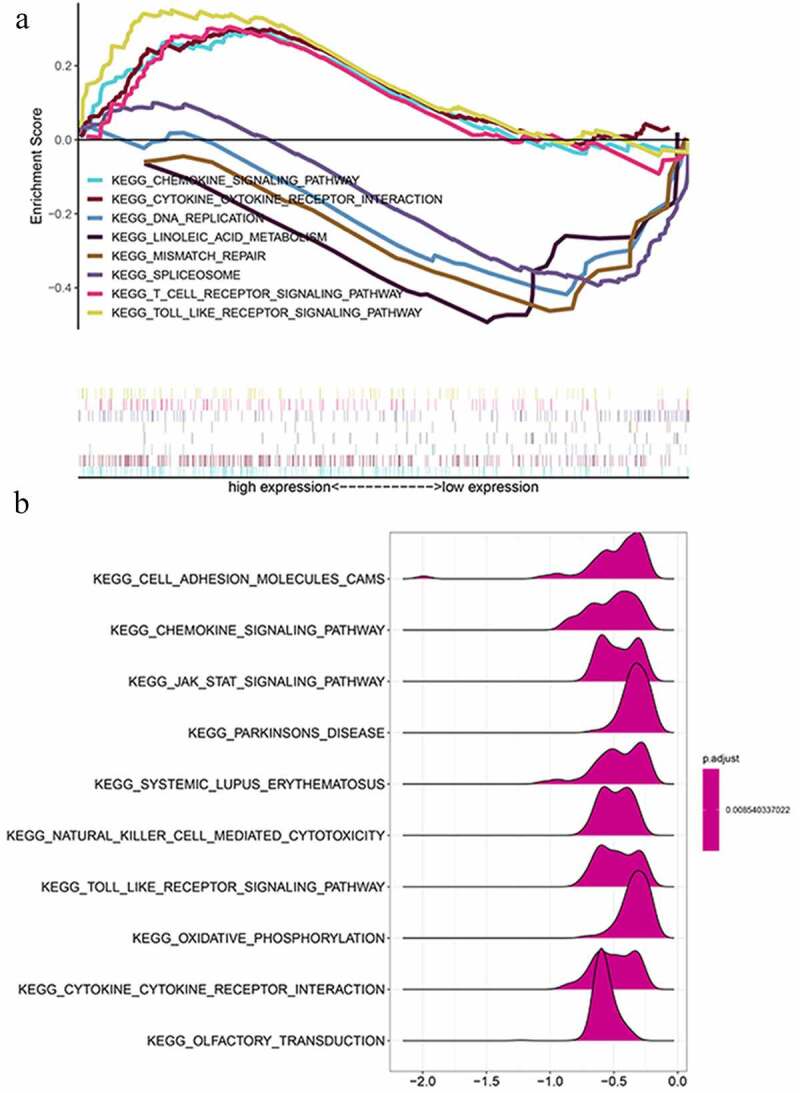
GSEA results and KEGG enrichment. (a) Top enriched KEGG pathways in the high risk group are represented by the curves above the x-axis in the graph. Top enriched KEGG pathways in the low risk group are represented by the curves below the x-axis in the graph (p-value < 0.05) in TCGA dataset. The names of enriched KEGG pathways are listed on the right side. (b) GSEA plots of KEGG Pathways in which the WTAP, KIAA1429 and HNRNPA2B1 were co-enriched

**Figure 8.**
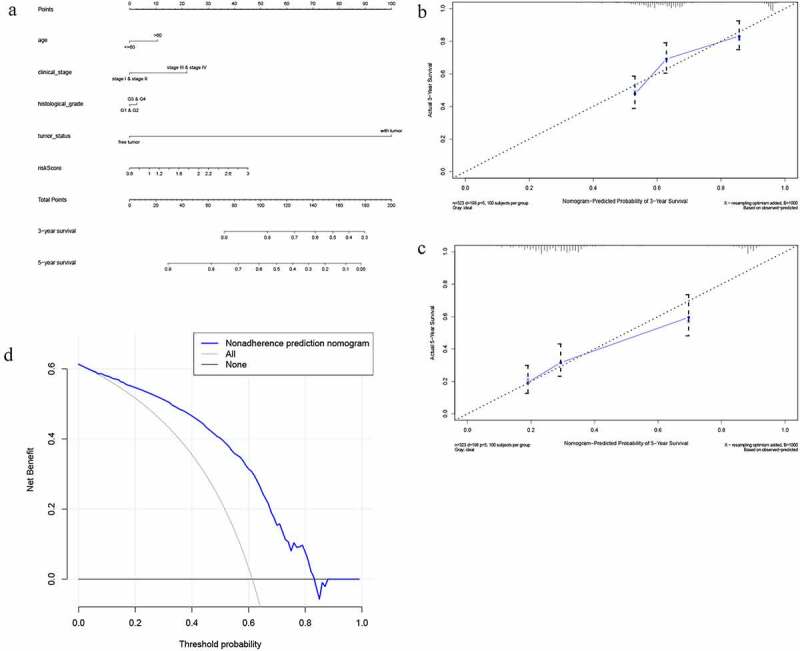
The nomogram to predict 3‐ or 5‐year OS in the entire set. (a) The nomogram for predicting proportion of patients with 3‐ or 5‐year OS. (b-c) The calibration plots for predicting patient 3‐ or 5‐year OS. Nomogram‐predicted probability of survival is plotted on the x‐axis; actual survival is plotted on the y‐axis. (d) DCA for assessment of the clinical utility of the nomogram. The x‐axis represents the percentage of threshold probability, and the y‐axis represents the net benefit. DCA: decision curve analysis; OS: overall survival

Two clusters of m6A RNA methylation regulators were associated with distinct OC clinical outcomes and clinicopathological features

With clustering stability increasing from k = 2 to 10, k = 2 seemed to be an adequate selection based on the expression similarity of m6A RNA methylation regulators (Figure 2a-c). Then, 379 OC samples were clustered into two subgroups in the TCGA dataset (cluster 1:216; cluser2:163). The two subgroups were named as cluster 1 and cluster 2 and clinicopathological features of the 2 two clusters were compared by k = 2 (Figure 2d). A significantly shorter OS was displayed in Cluster 1 than in Cluster 2 (Figure 2e).

Categories identified by consensus clustering are closely associated with the progression of OC

In order to understand the interactions among the 17 m6A RNA methylation regulators, the interaction (Figure 3a) and correlation (Figure 3b) among these regulators were analyzed. METTLE3 lies at the core of the network of m6A RNA methylation regulators. Its interactions and co-expressions with KIAA1429, METTLE14, WTAP, YTHDF1, YTHDF3, YTHDF2, and YTHDC1 were constructed and displayed in the String database. METTLE3 was also significantly correlated with YTHDC2 and YTHDF3. Three genes might co-work to regulate the progression of OC. The functional analysis of the two clusters was further performed. GSEA was functioned to show that the most relative GO terms were chemokine activity, macrophage chemotaxis, macrophage migration, monocyte chemotaxis, and tumor necrosis factor biosynthetic process in cluster 1 and cluster 2 (Figure 3c). The most relative pathways were allograft rejection, asthma, graft-versus-host disease, oxidative phosphorylation, and ribosome (Figure 4). The above findings suggest that the two categories identified by consensus clustering are closely associated with the progression of OC.

m6A RNA methylation regulators had prognostic significance

The prognostic ability of m6A RNA methylation regulators in OC was investigated. Univariate Cox regression analysis was conducted based on the expression levels of m6A RNA methylation regulators (Figure 5a). The results indicated that the three genes were significantly correlated with OS (P < 0.05). WTAP and KIAA1429 were two risky genes with HR > 1, while HNRNPA2B1 was a risky gene with HR < 1. To evaluate the ability of m6A RNA methylation regulators in predicting the clinical outcomes of OC, the LASSO Cox regression algorithm on three prognosis-associated genes was performed (Figure 5b-c), which were selected to construct the risk signature based on the minimum criteria. Coefficients obtained from LASSO algorithm were used to calculate the risk score: HNRNPA2B1*-0.01+ KIAA1429*0.085+ WTAP*0.03. The OC samples (n = 374) into low- and high-risk groups were separated based on the median risk score. The distribution of risk score, survival status, and the expression of three genes from each patient were also displayed (Figure 5d-f). Significant difference was observed in OS between the two groups (Figure 6a). ROC curves for 5-year survival were used to reveal the predictive performance of the three gene risk signatures. The 5-year AUC of the signature was 0.649, which was obviously higher than that of stage (AUC = 0.512), grade (AUC = 0.525) and age (AUC = 0.539) (Figure 6b). The results showed the three gene risk signatures had a stronger ability to predict OC survival than clinical factors.

### Prognostic value and clinical utility of three m6A regulators

**Figure 9.**
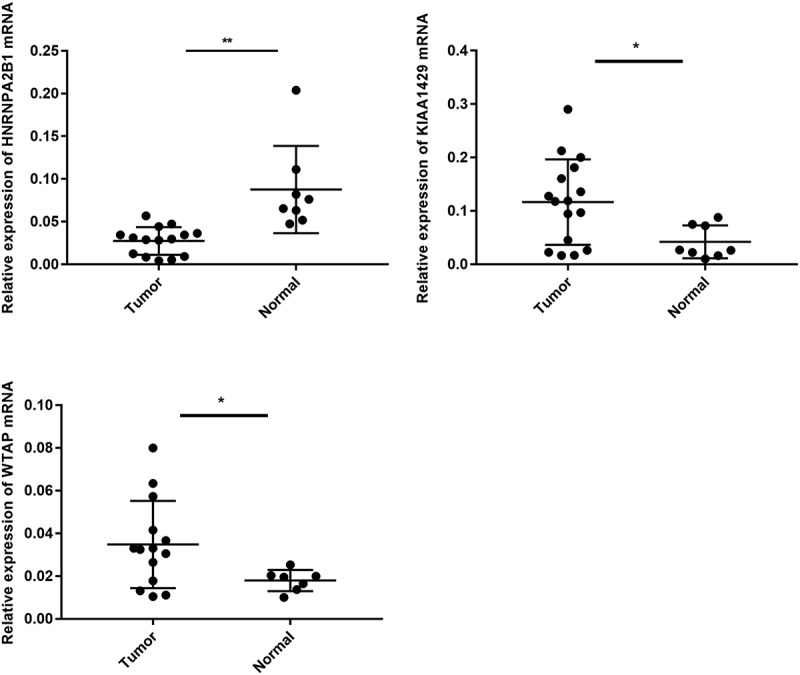
Expression level of WTAP, KIAA1429 and HNRNPA2B1 in clinical group

the TGGA dataset, univariate and multivariate regression models were constructed to identify whether the risk signature was an independent prognostic factor. Univariate analysis showed that tumor status and risk score were both correlated with OS (Figure 6c). Having absorbed three genes into the multivariate regression analysis, tumor status and risk score remained significantly correlated with OS (Figure 6d). Furthermore, the clinical features were associated with the three genes and Risk score (Table 1). It has also been found that the WTAP expression level is significantly different in different age groups (Figure 6e). The expression levels of HNRNPA2B1 in the TUMOR FREE group and the TUMOR group were also significantly different (figure 6f). Risk scores of patients in different age groups were also significantly different (Figure 6g). The stratification analysis was then performed based on grade, age, stage, and tumor status. Patients were stratified into Grade I/II and Grade III/IV subgroups and Stage I/II and Stage III/IV subgroups. As shown in **Supporting** Figure 9a, the prognosis of high-risk patients was significantly worse than that of low-risk patients in the Stage III/IV subgroup, which was consistent with the results of Grade II/IV subgroup (**Supporting** Figure 9b). However, there is no statistical significance in Stage I/II subgroup and Grade I/II subgroup. The prognostic ability of the three-gene signature combined with age and tumor status was also assessed. The patients were also stratified into different subgroups, including subgroups which is above 60 years and below 60 years. Interestingly, it has been revealed that high-risk patients in two subgroups were inclined to unfavorable OS (**Supporting** Figure 9c-e). Most of the immunity-related pathways were enriched in high-risk group, like T cell receptor signaling pathway, cytokine receptor interaction, and TOLL-like receptor signaling pathway. Most of the immunity-unrelated pathways were enriched in the low-risk group, like DNA replication and linoleic acid metabolism (Figure 7a). The three genes from the risk score model were co-enriched in cell adhesion molecule cams and chemokine signaling pathway (Figure 7b). The expression levels of three hub genes in 16 ovarian cancer clinical tissues and 8 normal ovarian tissues were also verified by the research (Figure 9). The results showed that the expression of HNRNPA2B1 was higher in normal ovarian tissues, and the expression of KIAA1429 was higher in ovarian cancer tissues, and both of them were consistent with our predicted trends. The expression level of WTAP was higher in ovarian cancer tissues, which was contrary to our prediction.

**Table 1.** Clinical significance of three prognosis-related genes

Gene	Age (≥60/<60)		Stage (I–II/III–IV)		Grade (1–2/3-4)		Tumor Status (with tumor/tumor free)	
	T	P	T	P	T	P	T	P
HNRNPA2B1	−0.165	0.869	1.49	0.154	−0.571	0.570	2.844	**0.005**
KIAA1429	−0.615	0.539	1.909	0.072	−1.268	0.209	0.082	0.935
WTAP	3.641	**3.264e-04**	1.337	0.198	1.218	0.230	−0.146	0.884

Bold values indicate *P < *0.05.

Note: t: t value of student’s t test; P: P‐value of student’s t test.

Association between three m6A regulators and immune infiltration

TCGA dataset was used to search the most significant tumor-infiltrating immune cells. Risk score was calculated to indicate the association between immune infiltration and three m6A regulators. Dendritic fraction, Macrophage fraction, and Neutrophil fraction have been discovered to be mostly enriched in high-risk group (**Supporting Figure 10A-C**).

A nomogram based on three m6A regulators

Encompassing age, stage, grade, tumor status, and risk score, a nomogram was constructed to predict the three-year or five-year OS of OC (Figure 8a). The calibration curve form Figure 8b-c suggests that the nomogram exhibited a performance as good as that of the Kaplan–Meier estimates. The C-index for this nomogram was 0.789 and became 0.773 after bootstrapping validation, showing its good discriminating ability. Meanwhile, DCA was created to estimate the clinical utility of the nomogram. The results of DCA showed that the nomogram containing three mRNAs’ signature had better prediction ability, with a threshold ranging from 2% to 83% (Figure 8d).

### Genetic information of the seventeen genes

The genetic alteration harbored in the 17 genes was analyzed with cBioPortal software. The network was exhibited, which is constructed by METTL3, HNRNPA2B1, HNRNPC, FMR1, and their 50 most associated neighbor genes (only four out of the 17 genes had a joint node, while the remaining three genes had no junctions and were not shown) (**Supporting Figure 11A**). **Supporting Figure 11B-C** illustrates that the 17 genes were altered in 471 (79%) of the 594 patients (606 in total); YTHDF1, WTAP, and ZC3H13 showed the most diverse alterations, including amplification, missense mutation etc.

## Discussion

Ovarian cancer is the most common gynecological malignancy. Most of the women have developed advanced stage when diagnosed [37]. Early diagnosis is critical to improve OC prognosis because the 5-year relative survival rate at the local stage is 93%. Specific biological diagnostic markers have been defined [38]. m6A modification has been implicated in mRNA turnover, localization, or translation [39–41]. As mainstream RNA regulators, m6A modulators have been widely proven to coordinate proteins related to coding functions in mRNA, and ultimately affecting tumors such as colorectal cancer, melanoma, etc. [42–47]. We hope to find m6a regulators related to ovarian cancer in batches. Therefore, it was reasonably speculated that m6A RNA methylation regulators are associated with ovarian cancer. It was also identified that two OC subgroups (Cluster 1 and Cluster 2) based on the expression of m6A RNA methylation regulators during the research. Two clusters were not only related to OC prognosis and clinicopathological features, but also related to some functional pathways, including graft-versus-host disease and oxidative phosphorylation. Coincidentally, these functional pathways have been found to regulate the development of OC. For example, Bay JO et al. found that an OC patient developed acute graft-versus-host disease and from this time her tumor diminished progressively [48]. Hänel M et al. also discovered a graft-versus-tumor effect in refractory ovarian cancer [49]. Pastò A et al. found oxidative phosphorylation in the stem cells from epithelial ovarian cancer patients [50]. Oxidative phosphorylation has also been validated as a therapeutic target for ovarian cancer [51].

METTL3 is the most widely studied writer. The experiments of Hua W et al. demonstrated that METTL3 promoted ovarian carcinoma growth and invasion through regulating AXL translation and epithelial to mesenchymal transition [3]. Cai X et al. demonstrated that HBXIP-elevated METTL3 expression and promoted the progression of breast cancer via inhibiting tumor suppressor let-7 g [52]. Vu LP et al. found that METTL3 curbed myeloid differentiation of normal hematopoietic and leukemia cells [53]. It has been identified that METTLE3 co-worked with others in OC development as the central gene in the network of m6A RNA methylation regulators. This conclusion has been proven in previous studies [54,55]. Studies have shown that N6-methyladenosine (m6A) is installed by the METTL3-METTL14-WTAP methyltransferase complex [56]. It has been identified that METTLE3 co-worked with others in OC development as the central gene in the network of m6A RNA methylation regulators. This conclusion has been proven in previous studies [57]. Wang Qiang and others found that the expression of METL3 promotes tumor angiogenesis and glycolysis in gastric cancer, and Mettl3 may be a cancer-promoting factor for gastric cancer [58]. Mettl3 has also been proven to promote the progression of cervical cancer, which is also a gynecological tumor [59].

Another writer associated with ovarian cancer is WTAP [60]. Consistently, our results showed that WTAP had a mutation rate of 16% and was highly expressed in patients aged over 60. The older the age, the greater the cumulative mutational load. The prognosis of bladder cancer and malignant glioma is also affected by WTAP [61,62]. Jing Wang et al. found that WTAP functions as an oncogenic factor that promotes the progression of ovarian cancer in which WTAP-HBS1L/FAM76A axis may be involved [63].

METTL14 is the main factor involved in aberrant m6A modification. Ma JZ et al. found that METTL14 as a writer suppressed the metastatic potential of hepatocellular carcinoma by modulating N6-methyladenosine-dependent primary microRNA processing [64]. In the study, METTL14 was lowly expressed in ovarian cancer samples and highly expressed in patients of Stage 1/2 subgroup and correlated with OC prognosis; thus, it is clear that the same M6A regulator also exerted different effects in different tumors. Mettl14 has also been shown to be related to breast cancer, colorectal cancer, and pancreatic cancer [65–67]

KIAA1429, METTL16, RBM15, and ZC3H13 were less studied in tumors. Qian JY et al. found that KIAA1429 acted as an oncogenic factor in breast cancer by regulating CDK1 in an N6-methyladenosine-independent manner [68]. KIAA1429 also has been proven to regulate cell proliferation by targeting c-Jun messenger RNA directly in gastric cancer [69] and participate in the migration and invasion of hepatocellular carcinoma [70]. ZC3H13 was found to suppress colorectal cancer proliferation and invasion [71]. ZC3H13 was also found to be Tumor Suppressor Genes in Breast Cancer [72]. We found that ZC3H13 showed a mutation rate of 18% and highly expressed in OC samples, and its expression level was negatively correlated with OC prognosis. It has been discovered that METTL16 was lowly expressed in OC tissues and was positively correlated with the prognosis via this study. METTL16 was lowly expressed in the samples collected from patients younger than 60. The same trend was shown in patients of stage III–IV subgroup and with tumor subgroup. These results support our hypothesis that METTL16 suppressed the development of OC. These findings indicate that increasing the level of m6A enrichment which was conducted by the writer can indeed alter the development of the tumor. However, a specific mechanism still needs to be tapped.

As complementary factors of writers, erasers also exert effects on a variety of tumors. Obesity is a high-risk factor for many tumors [73], and fat mass and obesity (FTO) are associated with obesity [74]. Akbari ME et al. found that FTO gene affected obesity and breast cancer through similar mechanisms [75]. FTO is associated with the occurrence and prognosis of gastric cancer [76]. Alkylation repair homolog protein 5 (ALKBH5) is also associated with pancreatic cancer [77], gastric cancer [78] and breast cancer [79]. Zhu H et al. found that ALKBH5 inhibited autophagy of epithelial ovarian cancer through regulating miR-7 and BCL-2 [80].

Three readers (YTHDF1, YTHDF2, and YTHDF3) were all highly expressed in OC samples and negatively related to prognosis. YTHDF1, YTHDF2, and HNRNPC have been intensely studied. YTH domain family 1 (YTHDF1) has a mutation rate of 27% and high expression rate in OC samples, which is associated with the poor prognosis of OS and DFS. YTHDF1 has been shown to be involved in the regulation of colorectal and pancreatic cancer [81,82]. Overexpression of YTHDF1 is associated with the poor prognosis of hepatocellular carcinoma [83]. In pancreatic cancer cells, YTHDF2 orchestrates epithelial–mesenchymal transition/proliferation dichotomy [82]. Li J et al. found that downregulation of N6-methyladenosine binding YTHDF2 protein mediated by miR-493-3p suppressed prostate cancer [84]. YTHDF2 also has a certain regulatory effect in lung cancer and gastric cancer [85,86]. HNRNPC can serve as a candidate biomarker for chemoresistance in gastric cancer [87]. Kleemann M et al. demonstrated that MiR-744-5p could induce cell death by directly targeting HNRNPC and NFIX in ovarian cancer [88]. The BRCA gene mutation is a feature of hereditary ovarian cancer. The samples were classified according to the presence of BRCA gene mutation [89], and it showed that the expression of FMR1 was related to BRCA gene mutation. Gleicher N et al. also found that BRCA/FMR1 had a correlation with ovarian cancer [90]. The mechanism of BRCA/FMR1 mutation causing ovarian cancer deserves further study.

A prognostic regression analysis found that HNRNPA2B1, KIAA1429, and WTAP have the strongest correlation with OC. Subsequently, OC patients were stratified into two subgroups with statistically different survival outcomes. In addition, univariate and multivariate Cox analyses identified the prognostic signature as an independent factor. In this study, due to the lack of an external validation cohort, to validate the prognostic performance of the 3-mRNA signature cannot be achieved. Thus, bootstrapping with 1,000 resamples was applied to internally validate the performance of three mRNA signature. The C-index for the internal validation was 0.773, indicating its good performance in clinical use. Moreover, a nomogram containing the 3-mRNA signature and other clinical features of ovarian cancer was built. The nomogram showed a moderate performance in predicting the survival of OC patients. Meanwhile, the results of DCA suggested that the nomogram showed better prediction ability, with a threshold ranging from 2% to 83%. Except WTAP, the role of both HNRNPA2B1 and KIAA1429 in ovarian cancer has not been thoroughly studied and can be used to direct further research. GSEA showed that the samples from the high-risk group were mainly enriched in immune-related pathways. Interestingly, the study showed that Dendritic fraction, Macrophage fraction, and Neutrophil fraction were related to the three m6A regulators. Surprisingly, there are experiments demonstrating that dendritic cell (DC) immunotherapy can induce anti-tumor T cell immunity [91]. Macrophage can regulate the progress of OC through multiple mechanisms like CD47 [92] and NF-κB activation [93]. Meta-analysis by Chen S et al. showed neutrophil-to-lymphocyte ratio is a potential prognostic biomarker in patients with ovarian cancer [94]. It can be seen that distorted immune microenvironment may induce tumors to some extent.

This research has the following shortcomings: 1. The model is not validated with external data. 2. Lack of verification of in vitro and in vivo experiments. Prospective clinical trials are necessary in the future to reconfirm the findings.

## Conclusion

This study analyzed the association between m6A regulators and clinical features of OC. The three selected m6A RNA methylation regulators (HNRNPA2B1, KIAA1429, and WTAP) showed high prognostic value for OC and were also enriched in the biological processes and signaling pathways that drive the malignant progression of OC. High-risk patients who had a dendritic fraction, macrophage fraction, and neutrophil fraction were also found in this study. Future clinical and experimental research is warranted to further verify the results of this study. In brief, this study provides novel markers for evaluating OC prognosis.

## Supplementary Material

Supplemental Material

## Data Availability

***Data availability statement*** The data and materials are available in The Cancer Genome Atlas (https://cancergenome.nih.gov/https://cancergenome.nih.gov/).

## Abbreviations

Ovarian cancer (OC) Search Tool for the Retrieval of Interacting Genes Database (STRING)
Receiver operating characteristic (ROC)
Gene set enrichment analysis (GSEA)
Decision curve analysis (DCA)
Kyoto Encyclopedia of Genes and Genomes (KEGG)
Area under the curve (AUC)
Hazard ratio (HR)

## Declaration

Ethics approval and consent to participate

The study was approved by the Ethics Committee of the Jiangsu Province Hospital of Chinese Medicine and samples were obtained with written informed consent from all patients, and all informed consent were conducted in accordance with the Declaration of Helsinki.

## Highlights

1.Three m6A regulatory genes were identified to predict OS of ovarian cancer patients.

2.The prognostic signature of three m6A regulatory genes are an independent prognostic factor.

3.The immune infiltrations are enriched in different risk groups.

## References

[1] Chen M, Wei L, Law CT, *et al*. RNA N6-methyladenosine methyltransferase-like 3 promotes liver cancer progression through YTHDF2-dependent posttranscriptional silencing of SOCS2. Hepatology. 2018;67(6):2254–2270. 29171881 10.1002/hep.29683

[2] Li J, Han Y, Zhang H, et al. Li B: the m6A demethylase FTO promotes the growth of lung cancer cells by regulating the m6A level of USP7 mRNA. Biochem Biophys Res Commun. 2019;512(3):479–485. 30905413 10.1016/j.bbrc.2019.03.093

[3] Hua W, Zhao Y, Jin X, et al. METTL3 promotes ovarian carcinoma growth and invasion through the regulation of AXL translation and epithelial to mesenchymal transition. Gynecol Oncol. 2018;151(2):356–365. 30249526 10.1016/j.ygyno.2018.09.015

[4] Chen M, Nie ZY, Wen XH, et al. m6A RNA methylation regulators can contribute to malignant progression and impact the prognosis of bladder cancer. Biosci Rep. 2019 ;39(12):BSR20192892 10.1042/BSR20192892 PMC6923333 31808521

[5] Ma S, Chen C, Ji X, et al. The interplay between m6A RNA methylation and noncoding RNA in cancer. J Hematol Oncol. 2019;12(1):121. 31757221 10.1186/s13045-019-0805-7 PMC6874823

[6] Ma Z, Li Q, Liu P, et al. METTL3 regulates m6A in endometrioid epithelial ovarian cancer independently of METTl14 and WTAP. Cell Biol Int. 2020;44(12):2524–2531. 32869897 10.1002/cbin.11459

[7] Li J, Wu L, Pei M, et al. YTHDF2, a protein repressed by miR-145, regulates proliferation, apoptosis, and migration in ovarian cancer cells. J Ovarian Res. 2020;13(1):111. 32948220 10.1186/s13048-020-00717-5 PMC7501604

[8] Fukumoto T, Zhu H, Nacarelli T, *et al*. N(6)-Methylation of Adenosine of FZD10 mRNA Contributes to PARP Inhibitor Resistance. Cancer Res. 2019;79(11):2812–2820. 30967398 10.1158/0008-5472.CAN-18-3592 PMC6548690

[9] Tomczak K, Czerwinska P, Wiznerowicz M. The Cancer Genome Atlas (TCGA): an immeasurable source of knowledge. Contemp Oncol (Pozn). 2015;19(1a):A68–77. 25691825 10.5114/wo.2014.47136 PMC4322527

[10] Lonsdale J, Thomas J, Salvatore M, et al. The Genotype-Tissue Expression (GTEx) project. Nat Genet. 2013;45(6):580–585 23715323 10.1038/ng.2653 PMC4010069

[11] Goldman MJ, Craft B, Hastie M, et al. Visualizing and interpreting cancer genomics data via the Xena platform. Nat Biotechnol. 2020;38(6):675–678. 32444850 10.1038/s41587-020-0546-8 PMC7386072

[12] Wagner GP, Kin K, Lynch VJ. Measurement of mRNA abundance using RNA-seq data: RPKM measure is inconsistent among samples. Theory Biosci. 2012;131(4):281–285. 22872506 10.1007/s12064-012-0162-3

[13] Ritchie ME, Phipson B, Wu D, et al. limma powers differential expression analyses for RNA-sequencing and microarray studies. Nucleic Acids Res. 2015;43(7):e47. 25605792 10.1093/nar/gkv007 PMC4402510

[14] Wilkerson MD, Hayes DN. ConsensusClusterPlus: a class discovery tool with confidence assessments and item tracking. Bioinformatics. 2010;26(12):1572–1573. 20427518 10.1093/bioinformatics/btq170 PMC2881355

[15] Keenan AB, Torre D, Lachmann A, et al. ChEA3: transcription factor enrichment analysis by orthogonal omics integration. Nucleic Acids Res. 2019;47(W1(W1):W212–w224. 31114921 10.1093/nar/gkz446 PMC6602523

[16] von Mering C, Huynen M, Jaeggi D, et al. STRING: a database of predicted functional associations between proteins. Nucleic Acids Res. 2003;31(1):258–261. 12519996 10.1093/nar/gkg034 PMC165481

[17] Subramanian A, Kuehn H, Gould J, et al. GSEA-P: a desktop application for Gene Set Enrichment Analysis. Bioinformatics. 2007;23(23):3251–3253. 17644558 10.1093/bioinformatics/btm369

[18] Zuo S, Wei M, Zhang H, et al. A robust six-gene prognostic signature for prediction of both disease-free and overall survival in non-small cell lung cancer. J Transl Med. 2019;17(1):152. 31088477 10.1186/s12967-019-1899-y PMC6515678

[19] Zhang M, Zhu K, Pu H, et al. An Immune-Related Signature Predicts Survival in Patients With Lung Adenocarcinoma. Front Oncol. 2019;9:1314. 31921619 10.3389/fonc.2019.01314 PMC6914845

[20] Loeb M, Bentley DW, Bradley S, *et al*. Development of minimum criteria for the initiation of antibiotics in residents of long-term-care facilities: results of a consensus conference. Infect Control Hosp Epidemiol. 2001 22;(2)120–124. 11232875 10.1086/501875

[21] Kivipelto M, Ngandu T, Laatikainen T, et al. Risk score for the prediction of dementia risk in 20 years among middle aged people: a longitudinal, population-based study. Lancet Neurol. 2006;5(9):735–741. 16914401 10.1016/S1474-4422(06)70537-3

[22] Kanehisa M, Sato Y, Kawashima M, et al. KEGG as a reference resource for gene and protein annotation. Nucleic Acids Res. 2016;44(D1(D1):D457–462. 26476454 10.1093/nar/gkv1070 PMC4702792

[23] Liu Y, Wu L, Ao H, et al. Prognostic implications of autophagy-associated gene signatures in non-small cell lung cancer. Aging (Albany NY). 2019;11(23):11440–11462. 31811814 10.18632/aging.102544 PMC6932887

[24] Gao J, Aksoy BA, Dogrusoz U, *et al*. Integrative analysis of complex cancer genomics and clinical profiles using the cBioPortal. Sci Signal. 2013;6(269):l1. 10.1126/scisignal.2004088 PMC4160307 23550210

[25] Liu Y, Yang Y, Luo Y, et al. Prognostic potential of PRPF3 in hepatocellular carcinoma. Aging (Albany NY). 2020;12(1):912–930. 31926109 10.18632/aging.102665 PMC6977647

[26] Li T, Fan J, Wang B, et al. TIMER: a Web Server for Comprehensive Analysis of Tumor-Infiltrating Immune Cells. Cancer Res. 2017;77(21):e108–e110. e108-e110. . 29092952 10.1158/0008-5472.CAN-17-0307 PMC6042652

[27] Huang C, Liu Z, Xiao L, et al. Clinical Significance of Serum CA125, CA19-9, CA72-4, and Fibrinogen-to-Lymphocyte Ratio in Gastric Cancer With Peritoneal Dissemination. Front Oncol. 2019;9:1159. 31750248 10.3389/fonc.2019.01159 PMC6848261

[28] Wu M, Li X, Zhang T, et al. Identification of a Nine-Gene Signature and Establishment of a Prognostic Nomogram Predicting Overall Survival of Pancreatic Cancer. Front Oncol. 2019;9:996. 31612115 10.3389/fonc.2019.00996 PMC6776930

[29] Pencina MJ, D’Agostino RB. Overall C as a measure of discrimination in survival analysis: model specific population value and confidence interval estimation. Stat Med. 2004;23(13):2109–2123. 15211606 10.1002/sim.1802

[30] Vickers AJ, Elkin EB. Decision curve analysis: a novel method for evaluating prediction models. Med Decis Making. 2006;26(6):565–574. 17099194 10.1177/0272989X06295361 PMC2577036

[31] Burkett K, Pickler R, Bowers K, et al. Disparities Affect Developmental Risk for Head Start Preschoolers. J Pediatr Nurs. 2020;54::86–92. 10.1016/j.pedn.2020.06.010 32682249

[32] Xu WH, Shi SN, Xu Y, et al. Prognostic implications of Aquaporin 9 expression in clear cell renal cell carcinoma. J Transl Med. 2019;17(1):363. 31703694 10.1186/s12967-019-2113-y PMC6842264

[33] Turner RB, Kojiro K, Shephard EA, et al. Review and Validation of Bayesian Dose-Optimizing Software and Equations for Calculation of the Vancomycin Area Under the Curve in Critically Ill Patients. Pharmacotherapy. 2018;38(12):1174–1183. 30362592 10.1002/phar.2191

[34] Conforti F, Pala L, Bagnardi V, et al. Sex-Based Heterogeneity in Response to Lung Cancer Immunotherapy: a Systematic Review and Meta-Analysis. J Natl Cancer Inst. 2019;111(8):772–781. 31106827 10.1093/jnci/djz094 PMC6695312

[35] Liu J, Li S, Liang J, et al. ITLNI identified by comprehensive bioinformatic analysis as a hub candidate biological target in human epithelial ovarian cancer. Cancer Manag Res. 2019;11::2379–2392. 10.2147/CMAR.S189784 PMC6438265 30988639

[36] Liu WL, Zhao JZ, Wang ZZ, et al. [Association between single nucleotide polymorphism of BARD1 gene and BRCA1 gene mutation in epithelial ovarian cancer]. Zhonghua Fu Chan Ke Za Zhi. 2017;52(6):403–410. 28647964 10.3760/cma.j.issn.0529-567X.2017.06.009

[37] Torre LA, Trabert B, DeSantis CE, et al. Ovarian cancer statistics, 2018. CA Cancer J Clin. 2018;68(4):284–296. 29809280 10.3322/caac.21456 PMC6621554

[38] Scaletta G, Plotti F, Luvero D, et al. The role of novel biomarker HE4 in the diagnosis, prognosis and follow-up of ovarian cancer: a systematic review. Expert Rev Anticancer Ther. 2017;17(9):827–839. 28756722 10.1080/14737140.2017.1360138

[39] Xiao W, Adhikari S, Dahal U, Chen YS, Hao YJ, Sun BF, Sun HY, Li A, Ping XL, Lai WY *et al*. Nuclear m(6)A Reader YTHDC1 Regulates mRNA Splicing. Mol Cell. 2016;61(4):507–519. 26876937 10.1016/j.molcel.2016.01.012

[40] Fustin JM,M,, Yamaguchi Y, Hida H, et al. RNA-methylation-dependent RNA processing controls the speed of the circadian clock. Cell. 2013;155(4):793–806. 24209618 10.1016/j.cell.2013.10.026

[41] Wang X, Zhao BS, Roundtree IA, et al. N(6)-methyladenosine Modulates Messenger RNA Translation Efficiency. Cell. 2015;161(6):1388–1399. 26046440 10.1016/j.cell.2015.05.014 PMC4825696

[42] García-Cárdenas JM, Guerrero S, López-Cortés A, et al. Post-transcriptional Regulation of Colorectal Cancer: a Focus on RNA-Binding Proteins. Front Mol Biosci. 2019;6:65. 31440515 10.3389/fmolb.2019.00065 PMC6693420

[43] Wurth L, Papasaikas P, Olmeda D, et al. UNR/CSDE1 Drives a Post-transcriptional Program to Promote Melanoma Invasion and Metastasis. Cancer Cell. 2016;30(5):694–707. 27908735 10.1016/j.ccell.2016.10.004

[44] Kakumani PK, Guitart T, Houle F, et al. CSDE1 attenuates microRNA-mediated silencing of PMEPA1 in melanoma. Oncogene. 2021;40(18):3231–3244. 10.1038/s41388-021-01767-9 33833398

[45] Wurth L, Gebauer F. RNA-binding proteins, multifaceted translational regulators in cancer. Biochim Biophys Acta. 2015;1849(7):881–886. 25316157 10.1016/j.bbagrm.2014.10.001

[46] Bisogno LS, Keene JD. RNA regulons in cancer and inflammation. Curr Opin Genet Dev. 2018;48::97–103. 10.1016/j.gde.2017.11.004 PMC6489128 29175729

[47] Culjkovic-Kraljacic B, Borden KLB. The Impact of Post-transcriptional Control: better Living Through RNA Regulons. Front Genet. 2018;9:512. 30455716 10.3389/fgene.2018.00512 PMC6230556

[48] Bay JO, Choufi B, Pomel C, et al. Potential allogeneic graft-versus-tumor effect in a patient with ovarian cancer. Bone Marrow Transplant. 2000;25(6):681–682. 10734306 10.1038/sj.bmt.1702206

[49] Hanel M, Bornhauser M, Muller J, et al. Evidence for a graft-versus-tumor effect in refractory ovarian cancer. J Cancer Res Clin Oncol. 2003;129(1):12–16. 12618895 10.1007/s00432-002-0399-1 PMC12161902

[50] Pasto A, Bellio C, Pilotto G, et al. Cancer stem cells from epithelial ovarian cancer patients privilege oxidative phosphorylation, and resist glucose deprivation. Oncotarget. 2014;5(12):4305–4319. 24946808 10.18632/oncotarget.2010 PMC4147325

[51] Nayak AP, Kapur A, Barroilhet L, et al. Oxidative Phosphorylation: a Target for Novel Therapeutic Strategies Against Ovarian Cancer. Cancers (Basel). 2018;**10**(9. 10.3390/cancers10090337 PMC6162441 30231564

[52] Cai X, Wang X, Cao C, et al. HBXIP-elevated methyltransferase METTL3 promotes the progression of breast cancer via inhibiting tumor suppressor let-7g. Cancer Lett. 2018;415:11–19. 10.1016/j.canlet.2017.11.018 29174803

[53] Vu LP, Pickering BF, Cheng Y, et al. The N(6)-methyladenosine (m(6)A)-forming enzyme METTL3 controls myeloid differentiation of normal hematopoietic and leukemia cells. Nat Med. 2017;23(11):1369–1376. 28920958 10.1038/nm.4416 PMC5677536

[54] Li X, Tang J, Huang W, et al. The M6A methyltransferase METTL3: acting as a tumor suppressor in renal cell carcinoma. Oncotarget. 2017;8(56):96103–96116. 29221190 10.18632/oncotarget.21726 PMC5707084

[55] Taketo K, Konno M, Asai A, et al. The epitranscriptome m6A writer METTL3 promotes chemo- and radioresistance in pancreatic cancer cells. Int J Oncol. 2018;52(2):621–629. 29345285 10.3892/ijo.2017.4219

[56] Sun HL, Zhu AC, Gao Y, et al. Stabilization of ERK-Phosphorylated METTL3 by USP5 Increases m(6)A Methylation. Mol Cell. 2020;80(4):633–647.e637. 33217317 10.1016/j.molcel.2020.10.026 PMC7720844

[57] Scholler E, Weichmann F, Treiber T, et al. Interactions, localization, and phosphorylation of the m(6)A generating METTL3-METTL14-WTAP complex. RNA. 2018;24(4):499–512. 29348140 10.1261/rna.064063.117 PMC5855951

[58] Wang Q, Chen C, Ding Q, et al. METTL3-mediated m(6)A modification of HDGF mRNA promotes gastric cancer progression and has prognostic significance. Gut. 2020;69(7):1193–1205. 31582403 10.1136/gutjnl-2019-319639

[59] Wang Q, Guo X, Li L, et al. N(6)-methyladenosine METTL3 promotes cervical cancer tumorigenesis and Warburg effect through YTHDF1/HK2 modification. Cell Death Dis. 2020;11(10):911. 33099572 10.1038/s41419-020-03071-y PMC7585578

[60] Yu HL, Ma XD, Tong JF, et al. WTAP is a prognostic marker of high-grade serous ovarian cancer and regulates the progression of ovarian cancer cells. Onco Targets Ther. 2019;12::6191–6201. 10.2147/OTT.S205730 PMC6689666 31496724

[61] Chen L, Wang X. Relationship between the genetic expression of WTAP and bladder cancer and patient prognosis. Oncol Lett. 2018;16(6):6966–6970. 30546429 10.3892/ol.2018.9554 PMC6256415

[62] Xi Z, Xue Y, Zheng J, et al. WTAP Expression Predicts Poor Prognosis in Malignant Glioma Patients. J Mol Neurosci. 2016;60(2):131–136. 27370540 10.1007/s12031-016-0788-6

[63] Wang J, Xu J, Li K, et al. Identification of WTAP-related genes by weighted gene co-expression network analysis in ovarian cancer. J Ovarian Res. 2020;13(1):119. 32998774 10.1186/s13048-020-00710-y PMC7528330

[64] Ma JZ, Yang F, Zhou CC, et al. METTL14 suppresses the metastatic potential of hepatocellular carcinoma by modulating N(6) -methyladenosine-dependent primary MicroRNA processing. Hepatology. 2017;65(2):529–543. 27774652 10.1002/hep.28885

[65] Wang M, Liu J, Zhao Y, et al. Upregulation of METTL14 mediates the elevation of PERP mRNA N(6) adenosine methylation promoting the growth and metastasis of pancreatic cancer. Mol Cancer. 2020;19(1):130. 32843065 10.1186/s12943-020-01249-8 PMC7446161

[66] Sun T, Wu Z, Wang X, et al. LNC942 promoting METTL14-mediated m(6)A methylation in breast cancer cell proliferation and progression. Oncogene. 2020;39(31):5358–5372. 32576970 10.1038/s41388-020-1338-9

[67] Chen X, Xu M, Xu X, et al. METTL14-mediated N6-methyladenosine modification of SOX4 mRNA inhibits tumor metastasis in colorectal cancer. Mol Cancer. 2020;19(1):106. 32552762 10.1186/s12943-020-01220-7 PMC7298962

[68] Qian JY, Gao J, Sun X, et al. KIAA1429 acts as an oncogenic factor in breast cancer by regulating CDK1 in an N6-methyladenosine-independent manner. Oncogene. 2019;38(33):6123–6141. 31285549 10.1038/s41388-019-0861-z

[69] Miao R, Dai CC, Mei L, et al. KIAA1429 regulates cell proliferation by targeting c-Jun messenger RNA directly in gastric cancer. J Cell Physiol. 2020;235(10):7420–7432. 32052427 10.1002/jcp.29645

[70] Cheng X, Li M, Rao X, et al. KIAA1429 regulates the migration and invasion of hepatocellular carcinoma by altering m6A modification of ID2 mRNA. Onco Targets Ther. 2019;12::3421–3428. 10.2147/OTT.S180954 PMC6510231 31118692

[71] Zhu D, Zhou J, Zhao J, et al. ZC3H13 suppresses colorectal cancer proliferation and invasion via inactivating Ras-ERK signaling. J Cell Physiol. 2019;234(6):8899–8907. 30311220 10.1002/jcp.27551

[72] Gong PJ, Shao YC, Yang Y, et al. Analysis of N6-Methyladenosine Methyltransferase Reveals METTL14 and ZC3H13 as Tumor Suppressor Genes in Breast Cancer. Front Oncol. 2020;10:578963. 33363011 10.3389/fonc.2020.578963 PMC7757663

[73] Deng X, Su R, Stanford S, et al. Critical Enzymatic Functions of FTO in Obesity and Cancer. Front Endocrinol (Lausanne). 2018;9:396. 30105001 10.3389/fendo.2018.00396 PMC6077364

[74] Zhao X, Yang Y, Sun BF, et al. FTO and obesity: mechanisms of association. Curr Diab Rep. 2014;14(5):486. 24627050 10.1007/s11892-014-0486-0

[75] Akbari ME, Gholamalizadeh M, Doaei S, et al. FTO Gene Affects Obesity and Breast Cancer Through Similar Mechanisms: a New Insight into the Molecular Therapeutic Targets. Nutr Cancer. 2018;70(1):30–36. 29220587 10.1080/01635581.2018.1397709

[76] Xu D, Shao W, Jiang Y, et al. FTO expression is associated with the occurrence of gastric cancer and prognosis. Oncol Rep. 2017;38(4):2285–2292. 28849183 10.3892/or.2017.5904

[77] Cho SH, Ha M, Cho YH, et al. ALKBH5 gene is a novel biomarker that predicts the prognosis of pancreatic cancer: a retrospective multicohort study. Ann Hepatobiliary Pancreat Surg. 2018;22(4):305–309. 30588520 10.14701/ahbps.2018.22.4.305 PMC6295372

[78] Zhang J, Guo S, Piao HY, et al. ALKBH5 promotes invasion and metastasis of gastric cancer by decreasing methylation of the lncRNA NEAT1. J Physiol Biochem. 2019;75(3):379–389. 31290116 10.1007/s13105-019-00690-8 PMC6728298

[79] Zhang C, Samanta D, Lu H, et al. Hypoxia induces the breast cancer stem cell phenotype by HIF-dependent and ALKBH5-mediated m(6) A-demethylationof NANOG mRNA. *Proceedings of the National Academy of Sciences of the United States of America* 2016, 113(14):E2047–2056. 10.1073/pnas.1602883113 PMC4833258 27001847

[80] Zhu H, Gan X, Jiang X, et al. ALKBH5 inhibited autophagy of epithelial ovarian cancer through miR-7 and BCL-2. J Exp Clin Cancer Res. 2019;38(1):163. 30987661 10.1186/s13046-019-1159-2 PMC6463658

[81] Nishizawa Y, Konno M, Asai A, et al. Oncogene c-Myc promotes epitranscriptome m(6)A reader YTHDF1 expression in colorectal cancer. Oncotarget. 2018;9(7):7476–7486. 29484125 10.18632/oncotarget.23554 PMC5800917

[82] Chen J, Sun Y, Xu X, et al. YTH domain family 2 orchestrates epithelial-mesenchymal transition/proliferation dichotomy in pancreatic cancer cells. Cell Cycle (Georgetown, Tex). 2017;16(23):2259–2271. 10.1080/15384101.2017.1380125 PMC5788481 29135329

[83] Zhao X, Chen Y, Mao Q, et al. Overexpression of YTHDF1 is associated with poor prognosis in patients with hepatocellular carcinoma. Cancer Biomarkers. 2018;21(4):859–868. 29439311 10.3233/CBM-170791 PMC13078334

[84] Li J, Meng S, Xu M, et al. Downregulation of N(6)-methyladenosine binding YTHDF2 protein mediated by miR-493-3p suppresses prostate cancer by elevating N(6)-methyladenosine levels. Oncotarget. 2018;9(3):3752–3764. 29423080 10.18632/oncotarget.23365 PMC5790497

[85] Sheng H, Li Z, Su S, et al. YTH domain family 2 promotes lung cancer cell growth by facilitating 6-phosphogluconate dehydrogenase mRNA translation. Carcinogenesis. 2020 ;41(5):541–550. 31504235 10.1093/carcin/bgz152

[86] Zhang J, Pi J, Liu Y, et al. [Knockdown of YTH N(6)-methyladenosine RNA binding protein 2 (YTHDF2) inhibits proliferation and promotes apoptosis in MGC-803 gastric cancer cells]. Xi Bao Yu Fen Zi Mian Yi Xue Za Zhi. 2017;33(12):1628–1634. 29382422

[87] Huang H, Han Y, Zhang C, et al. HNRNPC as a candidate biomarker for chemoresistance in gastric cancer. Tumour Biol. 2016;37(3):3527–3534. 26453116 10.1007/s13277-015-4144-1

[88] Kleemann M, Schneider H, Unger K, et al. MiR-744-5p inducing cell death by directly targeting HNRNPC and NFIX in ovarian cancer cells. Sci Rep. 2018;8(1):9020. 29899543 10.1038/s41598-018-27438-6 PMC5998049

[89] Toss A, Tomasello C, Razzaboni E, et al. Hereditary ovarian cancer: not only BRCA 1 and 2 genes. Biomed Res Int. 2015;2015:341723. 26075229 10.1155/2015/341723 PMC4449870

[90] Gleicher N, McAlpine JN, Gilks CB, et al. Absence of BRCA/FMR1 correlations in women with ovarian cancers. PLoS One. 2014;9(7):e102370. 25036526 10.1371/journal.pone.0102370 PMC4103842

[91] Drakes ML, Stiff PJ. Understanding dendritic cell immunotherapy in ovarian cancer. Expert Rev Anticancer Ther. 2016;16(6):643–652. 27078511 10.1080/14737140.2016.1178576

[92] Liu R, Wei H, Gao P, et al. CD47 promotes ovarian cancer progression by inhibiting macrophage phagocytosis. Oncotarget. 2017;8(24):39021–39032. 28380460 10.18632/oncotarget.16547 PMC5503592

[93] Cho U, Kim B, Kim S, et al. Pro-inflammatory M1 macrophage enhances metastatic potential of ovarian cancer cells through NF-kappaB activation. Mol Carcinog. 2018;57(2):235–242. 29024042 10.1002/mc.22750

[94] Chen S, Zhang L, Yan G, et al. Neutrophil-to-Lymphocyte Ratio Is a Potential Prognostic Biomarker in Patients with Ovarian Cancer: a Meta-Analysis. Biomed Res Int. 2017;2017:7943467. 28815182 10.1155/2017/7943467 PMC5549495

