# Supplementary material for: m6A RNA methylation regulators were associated with the malignancy and prognosis of ovarian cancer
Source: Bioengineered. 2021 Jun 30;12(1):3159–76. doi: 10.1080/21655979.2021.1946305 (PMC8806923; doi:10.1080/21655979.2021.1946305)
Supplement: Supplemental Material [file KBIE_A_1946305_SM2523.zip › supplementary/Table S1.docx]

**Table S1 Primer sequence of genes in qRT-PCR**

|  | Forward sequence | Reverse sequence |
| --- | --- | --- |
| WTAP | AGCGACTGAGTCCGCGATGGA | GCAGGATCCCTCATTACCACACAGT |
| kIAA1429 | CTTCCCAAGAAGGTTCGATTGA | TCAGACTCTCTTAGGCCAGTTAC |
| HNRNPA2B1 | TACTTTGAGCCCATTTCTCCTGA | GGAATACTGTCTACTGTTCGTCG |
