# Supplementary figures and images for: m6A RNA methylation regulators were associated with the malignancy and prognosis of ovarian cancer
Source: Bioengineered. 2021 Jun 30;12(1):3159–76. doi: 10.1080/21655979.2021.1946305 (PMC8806923; doi:10.1080/21655979.2021.1946305)

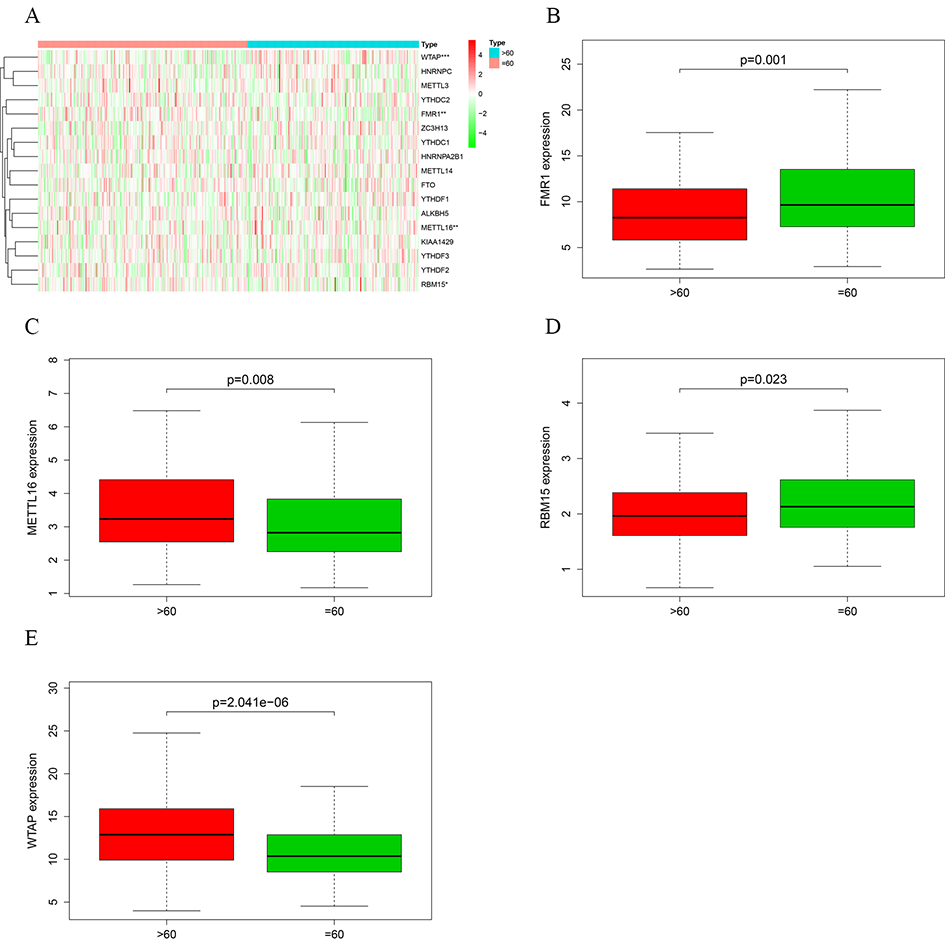

Supplement: Supplemental Material [file KBIE_A_1946305_SM2523.zip › supplementary/Supporting Figure 1.tif]

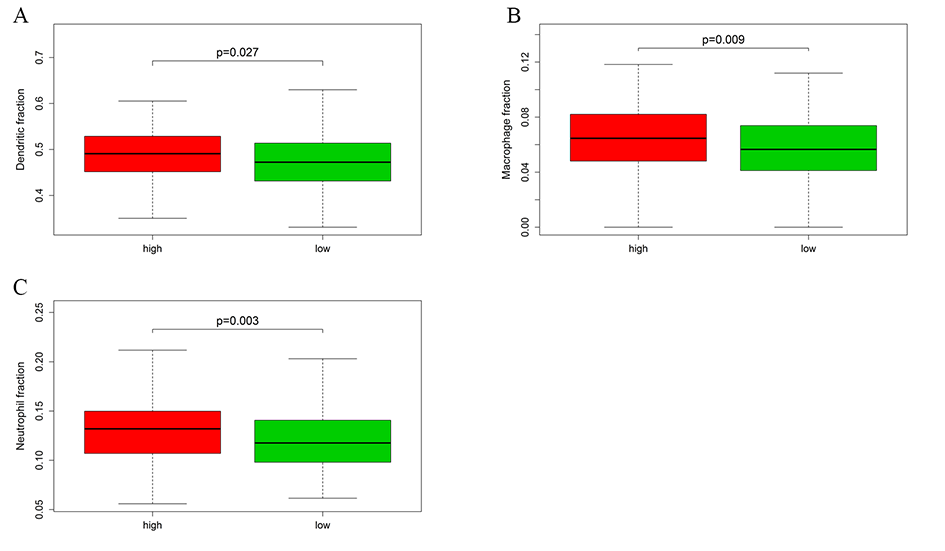

Supplement: Supplemental Material [file KBIE_A_1946305_SM2523.zip › supplementary/Supporting Figure 10.tif]

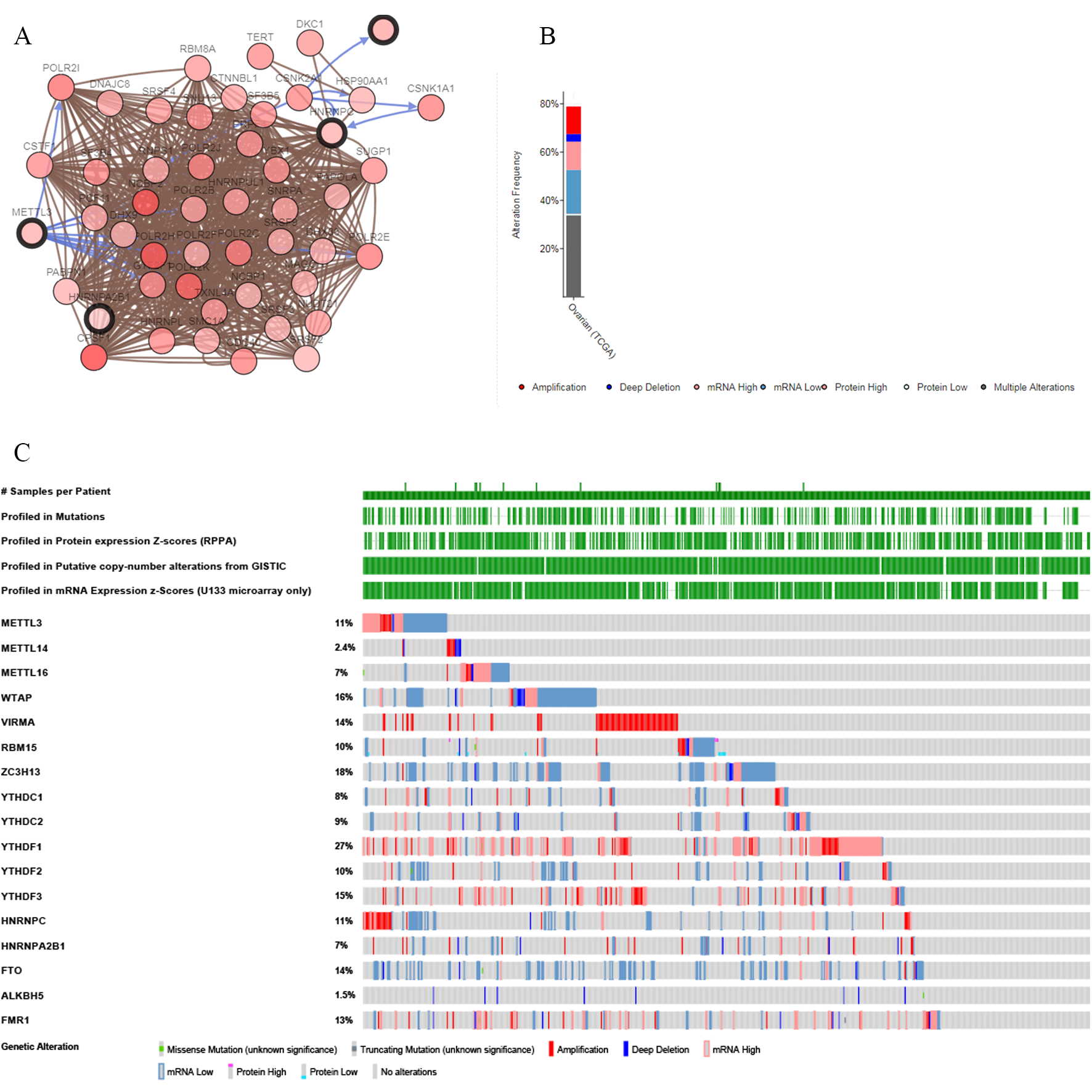

Supplement: Supplemental Material [file KBIE_A_1946305_SM2523.zip › supplementary/Supporting Figure 11.tif]

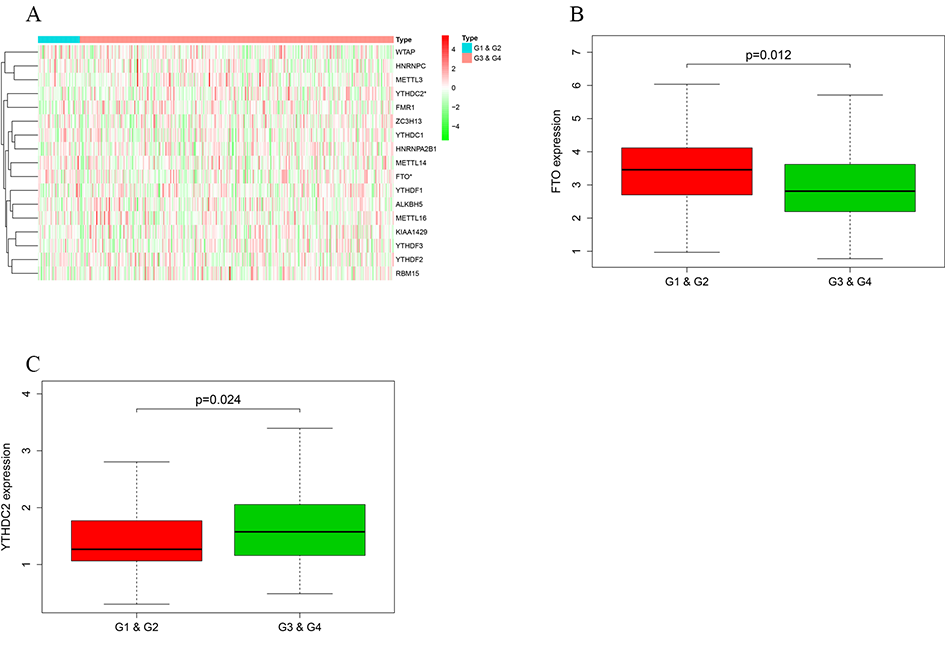

Supplement: Supplemental Material [file KBIE_A_1946305_SM2523.zip › supplementary/Supporting Figure 2.tif]

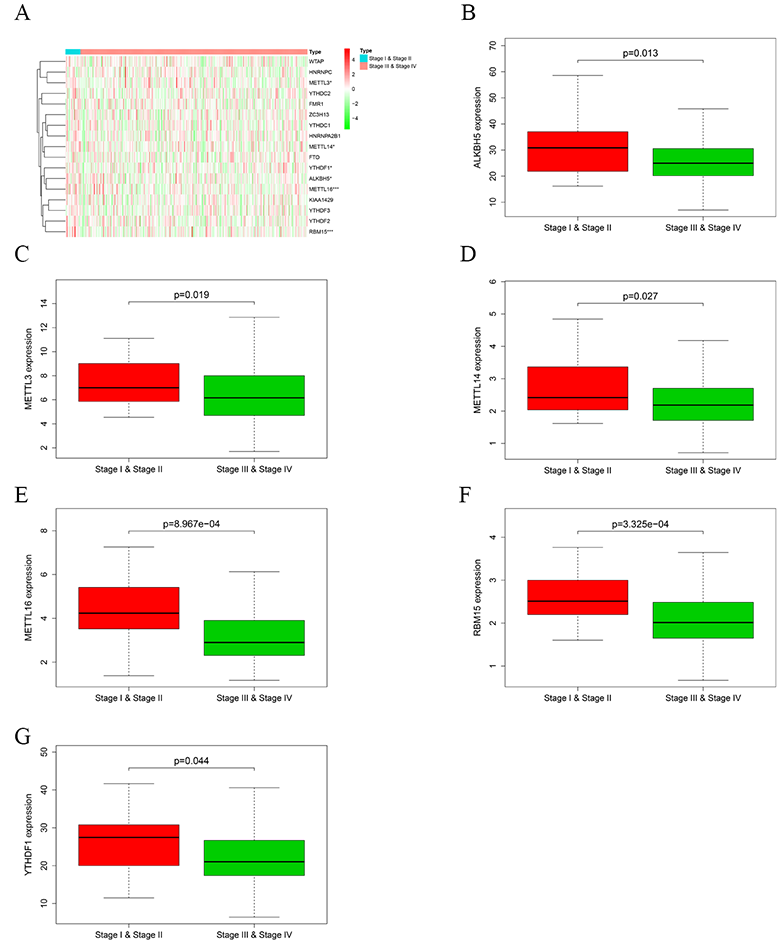

Supplement: Supplemental Material [file KBIE_A_1946305_SM2523.zip › supplementary/Supporting Figure 3.tif]

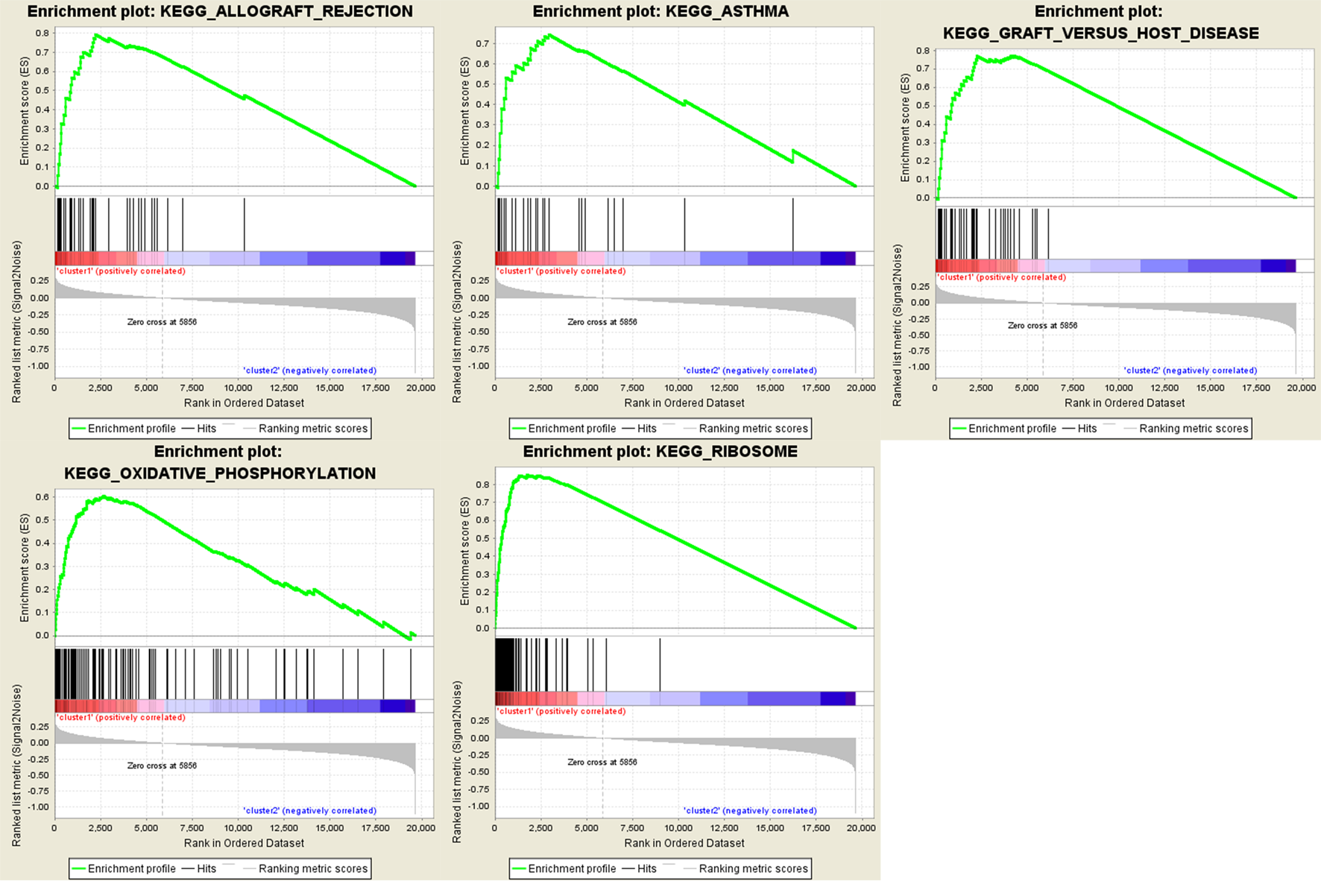

Supplement: Supplemental Material [file KBIE_A_1946305_SM2523.zip › supplementary/Supporting Figure 4.tif]

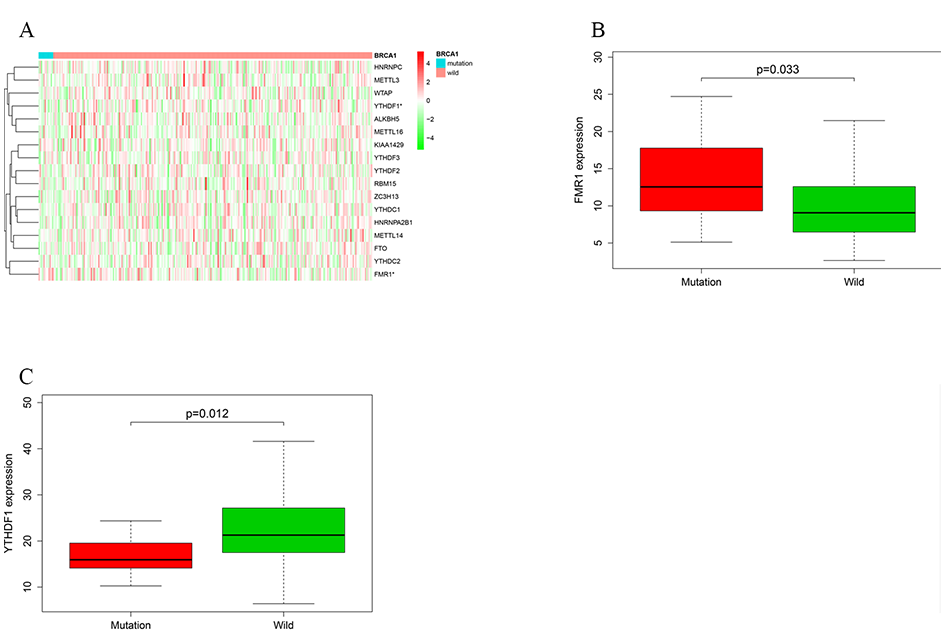

Supplement: Supplemental Material [file KBIE_A_1946305_SM2523.zip › supplementary/Supporting Figure 5.tif]

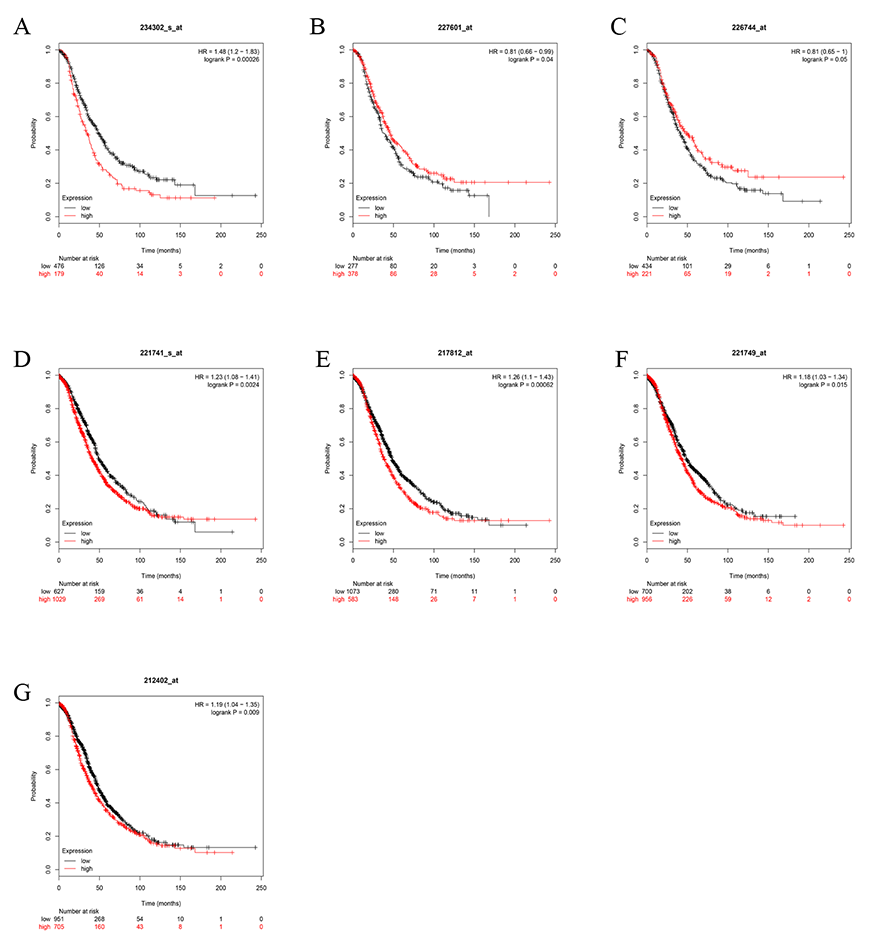

Supplement: Supplemental Material [file KBIE_A_1946305_SM2523.zip › supplementary/Supporting Figure 6.tif]

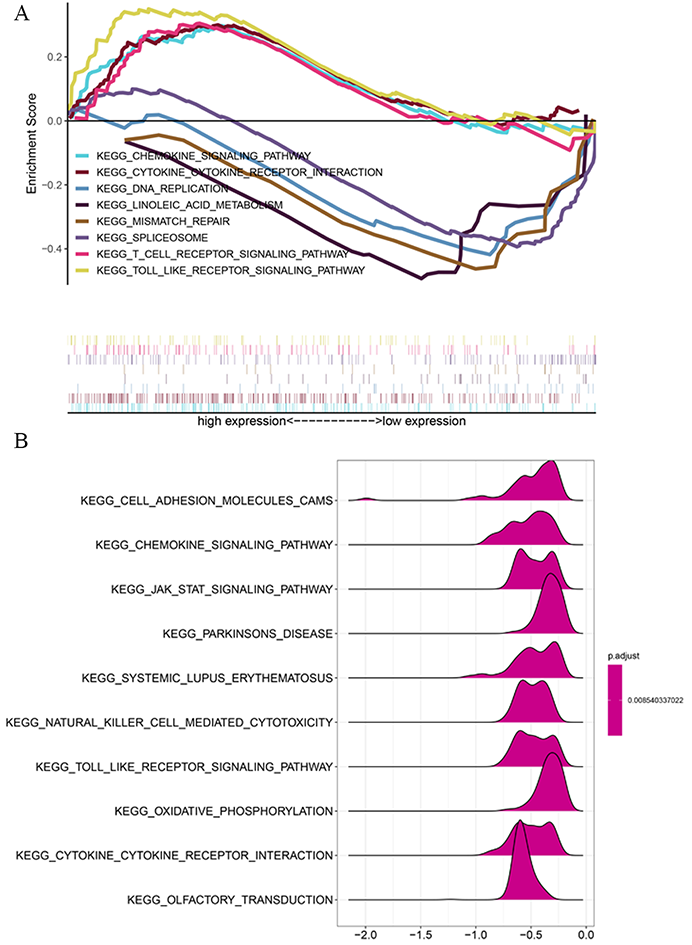

Supplement: Supplemental Material [file KBIE_A_1946305_SM2523.zip › supplementary/Supporting Figure 7.tif]

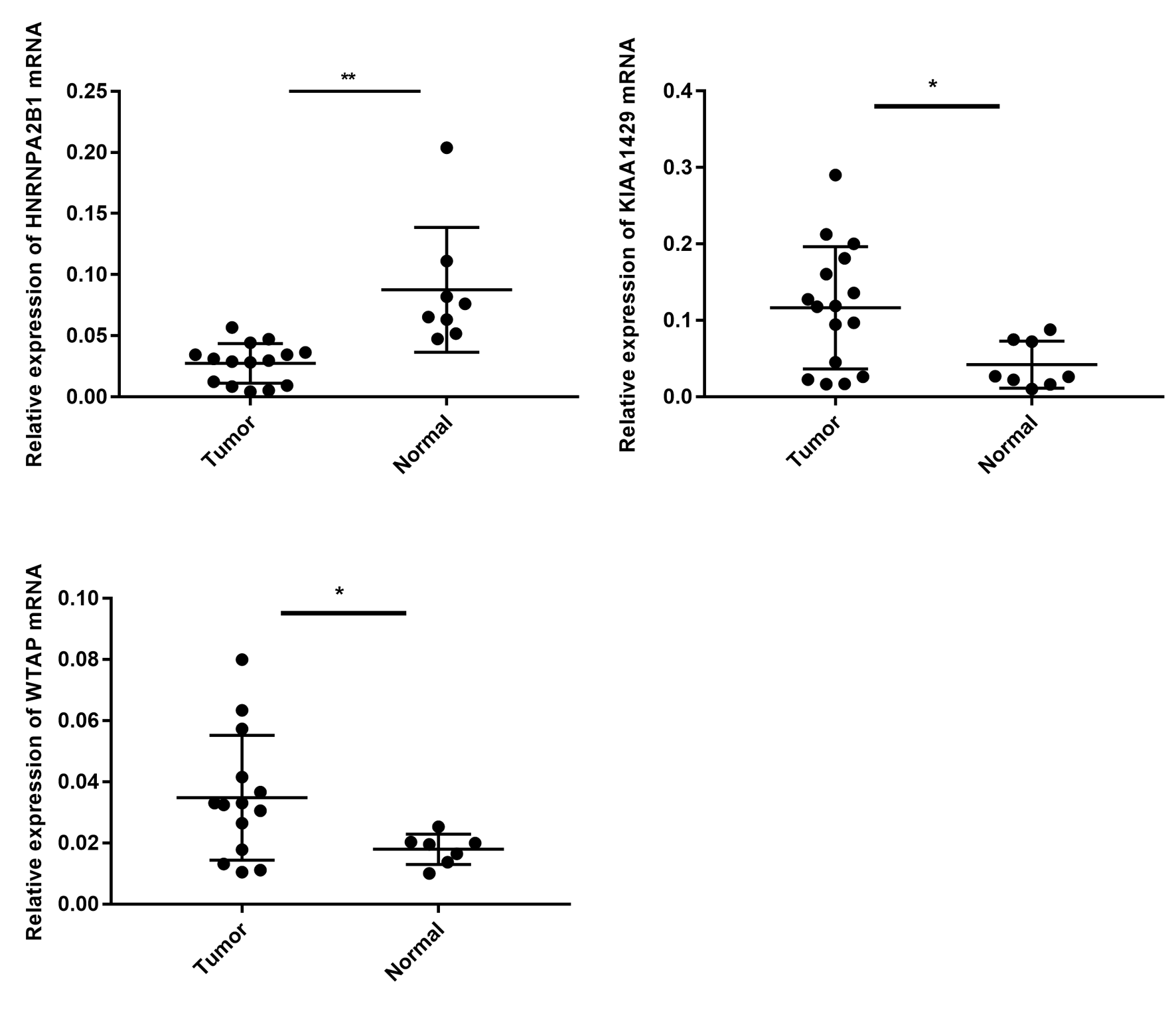

Supplement: Supplemental Material [file KBIE_A_1946305_SM2523.zip › supplementary/Supporting Figure 9.tif]
